# Peripheral complement C3 and C4 are associated with clinical features of schizophrenia

**DOI:** 10.3389/fpsyt.2026.1767438

**Published:** 2026-03-30

**Authors:** Marta Szwajca, Natalia Śmierciak, Beata Biesaga, Paulina Donicz, Krzysztof Szwajca, Maciej Pilecki

**Affiliations:** 1 Department of Psychiatry, Faculty of Medicine, Jagiellonian University Medical College, Kraków, Poland; 2 Faculty of Medicine, Andrzej Frycz Modrzewski Krakow University, Krakow, Poland

**Keywords:** anxiety, complement components, childhood trauma, cognitive performance, duration of untreated psychosis, schizophrenia, C3, C4

## Abstract

**Aim:**

Schizophrenia is a severe psychiatric disorder with heterogeneous outcomes; factors such as anxiety, childhood trauma, and duration of untreated psychosis (DUP) may influence symptom severity and disease progression. Growing evidence highlights immune dysregulation—particularly alterations in complement components C3 and C4—in the pathophysiology of schizophrenia; however, findings regarding peripheral complement levels and their clinical associations remain inconsistent.

**Method:**

Thirty-nine patients with schizophrenia underwent clinical assessment using the Childhood Trauma Questionnaire (CTQ), the Positive and Negative Syndrome Scale (PANSS), the State–Trait Anxiety Inventory (STAI), and the Montreal Cognitive Assessment (MoCA). Serum concentrations of C3 and C4 were measured at admission.

**Results:**

In exploratory analyses (nominal p-values), baseline C3 correlated with DUP (r=0.407, p=0.010) and length of hospitalization (r=0.353, p=0.028). Higher C3 was associated with greater symptom severity on PANSS-P_1_ (r=0.325, p=0.043) and PANSS-G_1_ (r=0.330, p=0.040), while C4 correlated with PANSS-G_1_ (r=0.322, p=0.045) and multiple PANSS domains after 12 weeks. C3 was associated with anxiety at baseline and after 3 months (STAI-T_1_: r=0.376, p=0.018; STAI-S_1_: r=0.372, p=0.020; STAI-T_2_: r=0.376, p=0.018; STAI-S_2_: r=0.419, p=0.009), whereas C4 correlated with STAI-T_1_ (r=0.361, p=0.024), STAI-S_1_ (r=0.342, p=0.033), and STAI-S_2_ (r=0.338, p=0.038). Higher C3 and C4 levels were associated with CTQ subscales. C3 correlated negatively with cognitive performance (MoCA_1_: r=–0.339, p=0.034). However, none of the associations survived Benjamini–Hochberg false discovery rate (BH-FDR) correction (all q>0.05).

**Conclusion:**

These exploratory, within-cohort findings suggest that peripheral complement markers relate to variation in clinical severity and illness-course indicators in schizophrenia. Replication in larger, controlled longitudinal studies is warranted.

## 1 Introduction

Schizophrenia is a devastating mental disorder associated with significant morbidity and an increased risk of premature death, with a lifetime morbid risk of approximately 0.7% worldwide (1, 2). It is the eighth leading cause of disability globally among individuals aged 15 to 44 (3). The disorder typically emerges during adolescence or early adulthood, a critical period of neurodevelopment (4). Its clinical course, presentation, and prognosis are highly heterogeneous, marked by frequent and unpredictable relapses that often lead to profound functional impairment (5). Schizophrenia is characterized by positive symptoms (e.g., hallucinations, delusions) and negative symptoms (e.g., diminished emotional expression, social withdrawal) (6). In addition, cognitive disturbances constitute a core feature of the disorder, affecting 75–80% of patients and encompassing broad deficits across multiple cognitive domains (7).

In addition to these well-established clinical characteristics, anxiety plays a significant role in schizophrenia, influencing its course, prognosis, and overall quality of life (8). Anxiety symptoms are highly prevalent in this population, affecting up to 65% of patients (9), and are associated with poorer clinical outcomes, including an increased risk of suicide and higher utilization of medical services. Despite its clinical relevance, anxiety in schizophrenia remains insufficiently investigated.

Another variable shown to influence the course of the illness is childhood abuse. A history of childhood trauma is recognized as a significant risk factor for psychotic symptoms and schizophrenia (10, 11). Numerous studies have demonstrated a strong association between childhood adversity and the development of psychosis, with an estimated odds ratio of approximately 3 (12). Among individuals with schizophrenia, childhood abuse has been identified as a significant predictor of poor treatment response (13), increased risk of suicide attempts and suicidal ideation (14), greater functional and social impairment (15), more severe psychotic symptomatology (16), and a higher number of hospitalizations (17). Although well established, the mechanisms through which early adversity shapes illness trajectory remain largely unclear.

Another significant prognostic factor in schizophrenia is the duration of untreated psychosis (DUP), defined as the period between the onset of psychosis and the initiation of adequate treatment (18). A comprehensive umbrella review provides robust evidence that longer DUP is associated with more severe positive and negative symptoms, as well as a reduced likelihood of remission (19).

Recent research provides compelling evidence for numerous connections between immunogenetic factors, inflammation, and major psychiatric disorders, including schizophrenia (20–24). Studies examining the role of immune processes in the onset, progression, complications, and prognosis of schizophrenia highlight the importance of diverse immunological mechanisms (25). Notably, there is robust evidence supporting a significant association between schizophrenia and dysregulation of the complement system (26).

The complement system, a central component of innate immunity and a bridge to adaptive immune responses, consists of soluble proteins, membrane-bound receptors, and regulatory molecules that maintain homeostasis, modulate inflammation, and defend against pathogens (27–30). Complement activation occurs through the classical, lectin, and alternative pathways, which differ in recognition mechanisms but converge on activation of the central complement protein C3 (28). This activation triggers downstream events, including antibody production, opsonization, induction of inflammation, activation of inflammasomes, cytokine secretion, immune cell activation, cell lysis, and clearance of immune complexes (28). While complement activation has protective functions, excessive or chronic activation may exert harmful effects through sustained release of pro-inflammatory mediators that damage cells and tissues (31).

Beyond its immunological role, the complement system performs crucial functions in the central nervous system (CNS), contributing to development, homeostasis, and regeneration (32). Complement signaling is involved in neural tube closure, progenitor cell proliferation and differentiation (33), and neuronal migration in the developing cortex (34), with impaired migration reported in schizophrenia (35). Complement-dependent microglial synapse elimination is a fundamental mechanism shaping synaptic plasticity (36–38). Notably, aberrant synapse elimination—reflected in dendritic spine abnormalities (39, 40) and reduced cortical thickness (41, 42)—has been documented in schizophrenia, consistent with the longstanding hypothesis of excessive synaptic pruning (43).

Persuasive evidence from the landmark study by Sekar et al. (2016) demonstrated that C4A alleles increase schizophrenia risk by mediating excessive synaptic pruning during postnatal development, and elevated C4A expression has been observed across cortical regions of individuals with schizophrenia (44). Additional studies show that C4 copy number variations are linked to neuropil contraction and reduced formation (45). Experimental models support these findings: C4 overexpression in mouse prefrontal cortex neurons disrupts dendritic spine development, produces hypoconnectivity, and induces social deficits (46), whereas C4A overexpression increases microglial engulfment and reduces synapse density, accompanied by abnormal social behavior, working memory deficits, and anxiety-like behaviors (47). Genetic studies also implicate C3 polymorphisms in schizophrenia risk (48), collectively suggesting that altered C3 and C4 expression may directly contribute to the disorder’s pathogenesis.

However, a review and meta-analysis (49) found no significant differences in serum or plasma C3 and C4 levels between patients with schizophrenia and healthy controls. This inconsistency may reflect substantial heterogeneity across studies, including clinical characteristics and illness stage. Similarly, findings regarding associations between complement proteins and symptomatology are conflicting: some studies report elevated C4 (50–52) or C3 levels (50, 52), others report decreased C3 (51, 53), while others find no associations (54, 55). More recent evidence further underscores this heterogeneity and suggests that peripheral complement alterations may depend on clinical subtype and illness phase. Göker et al. (2023) reported significantly higher serum C3 and C4 levels in treatment-resistant schizophrenia compared with patients in remission and healthy controls, with remission levels comparable to controls, alongside only weak positive associations between C3 and C4 concentrations and the severity of clinical symptoms (56). In a first-episode, drug-naïve sample, Cao et al. (2023) likewise observed elevated serum C3 and C4 levels versus controls, although only C3 showed a weak positive association with positive symptom severity (57). Yu et al. (2023) reported higher plasma C4 (but not C3) levels in drug-naïve first-episode schizophrenia; within patients, higher C3 levels were associated with poorer logical memory, whereas higher C4 levels were associated with cortical thinning (58). In contrast, Savukoski et al. (2024) found no differences in serum C4 between acutely ill unmedicated patients and matched controls, and no associations between C4 and the severity of clinical symptoms; C4 levels also did not change significantly after 6 weeks of treatment (59). Overall, these mixed findings indicate that the direction and clinical relevance of peripheral C3 and C4 alterations in schizophrenia remain uncertain, supporting the need for further studies.

Despite growing evidence for complement involvement in schizophrenia, its associations with clinical symptoms, cognitive functioning, and illness progression remain unclear. Although previous studies demonstrate links between anxiety and inflammation (60), and consistently report elevated inflammatory markers among individuals with a history of childhood trauma (61), the specific role of complement in anxiety—particularly within schizophrenia—remains poorly understood. Moreover, the relationship between childhood trauma and complement components in adulthood has been rarely investigated. In this study, we aimed to examine the role of complement proteins C3 and C4 in psychotic decompensation in schizophrenia, with particular focus on symptom severity and illness progression. Understanding the involvement of these components in psychotic decompensation may provide valuable insight into the immunological mechanisms underlying schizophrenia.

### 1.1 Research hypotheses

Based on previous evidence indicating heterogeneous but potentially clinically relevant alterations of peripheral complement components in schizophrenia, we examined the two measured complement proteins, C3 and C4, and formulated the following hypotheses. First, we hypothesized that higher complement component levels at hospital admission would be associated with greater severity of clinical symptoms, both at admission and after three months of hospitalization. Second, we hypothesized that elevated complement component levels would be associated with indicators of illness progression, including longer duration of untreated psychosis and prolonged hospitalization. Third, we hypothesized that higher complement component levels would be associated with higher state and trait anxiety at admission and after three months of hospitalization. Fourth, we hypothesized that greater exposure to childhood trauma would be associated with higher complement component levels. Finally, we hypothesized that higher complement component levels would be associated with poorer cognitive performance.

## 2 Materials and methods

### 2.1 Study participants

The study included 39 patients aged 15–38 years who were experiencing psychotic decompensation and were admitted to the inpatient wards of the Adult, Child, and Adolescent Psychiatry Clinical Department at the University Hospital in Krakow. In this study, psychotic decompensation was defined as an acute exacerbation of psychotic symptoms (e.g., increased severity of positive symptoms or marked behavioural disorganization) resulting in inpatient psychiatric admission and the initiation or intensification of antipsychotic treatment. All participants were diagnosed with schizophrenia (F20) based on the 10th revision of the International Statistical Classification of Diseases and Related Health Problems (ICD-10) (62), with the diagnosis independently confirmed by two psychiatrists.

Informed written consent was obtained from all participants. For those under 18 years of age, additional consent was provided by their legal guardians. Participants received a detailed protocol outlining the study’s description and plan and retained the right to withdraw at any stage. However, none of the participants exercised this option.

Recruitment was conducted after the study protocol was approved by the Bioethics Committee of Jagiellonian University (KBET 122.6120.23.2016). During the recruitment process, all patients were thoroughly assessed to ensure they met the inclusion criteria and did not meet any of the exclusion criteria.

The exclusion criteria were as follows: inability to provide informed consent; intellectual disability; hospitalization without the patient’s consent; substance abuse within three months prior to admission; the presence of affective symptoms; autoimmune diseases; acute inflammatory conditions; active or past oncological diseases; chronic terminal illnesses; cardiovascular disorders; a history of thyroid dysfunction, diabetes, or central nervous system (CNS) disorders (e.g., Alzheimer’s disease or Parkinson’s disease).

### 2.2 Clinical evaluation

Demographic and clinical data were collected from each patient, including the onset of the first psychotic symptoms and the duration of untreated psychosis (DUP). DUP was defined as the time from the onset of the first positive symptoms—regardless of the number of psychotic episodes—to the initiation of effective treatment during the first episode of psychosis, based on the earliest available medical records.

The psychiatric assessment was complemented with several standardized instruments, including the Childhood Trauma Questionnaire (CTQ), the Positive and Negative Syndrome Scale (PANSS), the State–Trait Anxiety Inventory (STAI), and the Montreal Cognitive Assessment (MoCA). PANSS, STAI, and MoCA assessments were conducted at two time points: at baseline (T1), during the initial hospitalization period, and after the 12-week treatment period (T2). PANSS was administered by a trained and clinically experienced psychiatrist, whereas CTQ, STAI, and MoCA were administered by a qualified psychologist.

#### 2.2.1 PANSS

PANSS (63) is the most established tool for the assessment of schizophrenia psychopathology (64, 65). PANSS is a 30-item, clinician-rated instrument that consists of three subscales measuring the severity of:

- Positive Symptoms (PANSS P) – seven items (e.g., *delusions, hallucinations*).
- Negative Symptoms (PANSS N) – seven items (e.g., *blunted affect, emotional withdrawal*), and
- General Psychopathology (PANSS G) – 16 items (e.g., *poor impulse control, tension*).

Each item is rated on a 7-point scale from 1 (none) to 7 (most severe). The total PANSS score (PANSS T) is the sum of the scores from the three subscales, ranging from 30 to 210 points, with higher scores indicating greater symptom severity.

PANSS assessments were conducted at baseline and 12 weeks later.

#### 2.2.2 MoCA

MoCA (66) is a cognitive test designed to assess mild cognitive impairment. Its high sensitivity and specificity have made it widely utilized across diverse clinical settings for detecting cognitive deficits (67). MoCA evaluates various domains of cognitive function: attention, concentration and working memory, executive functions, short term memory, language, visuospatial skills, and orientation to time and place. All of these cognitive domains are also recognized as being impaired in schizophrenia. MoCA test has a maximum score of 30 points, with a score of 26 or higher indicating normal cognitive function (66). MoCA proved to be a useful and sensitive instrument for determining neurocognitive performance in schizophrenia (67–74). Patients were assessed using MoCA at baseline and again 12 weeks later. To address the repetition of measurements, two equivalent versions of MoCA test, adapted into Polish, were employed (75).

#### 2.2.3 STAI

STAI is a self-report questionnaire that measures both state (STAI-S) and trait anxiety (STAI-T) (76). It is considered the gold standard for assessing anxiety (77) and is the most frequently used measure of state and trait non–disorder-specific anxiety. State anxiety indicates how the respondents feel at a particular moment in time and refers to a transient emotional condition that is characterized by subjective, consciously perceived feelings of tension and apprehension and heightened autonomic nervous system activity, whereas the trait anxiety shows how one generally feels and refers to relatively stable individual differences in anxiety proneness, means the tendency to react to situations perceived as threatening with elevations in state anxiety intensity (78).

Each of the two subscales consists of 20 items. Items are summed for each scale and converted into scores ranging from 20 to 80, with higher scores indicating greater anxiety severity. The Polish adaptation of STAI was used (79). STAI was administered to patients at baseline and again 12 weeks later.

#### 2.2.4 CTQ

CTQ (80, 81) is a 28-item retrospective self-report instrument used to assess exposure to childhood abuse and neglect. It is one of the most widely utilized and well-validated measures of childhood maltreatment (82, 83) and has emerged as a leading tool in this field (84).

CTQ evaluates five specific domains: sexual abuse (CTQ-SA), physical abuse (CTQ-PA), emotional abuse (CTQ-EA), physical neglect (CTQ-PN), and emotional neglect (CTQ-EN). The combined scores from these five subscales yield a total score (CTQ-Total). Each item is rated on a 5-point Likert scale ranging from 1 (never true) to 5 (very often true), with subscale scores ranging from 5 to 25 and a total score ranging from 25 to 125.

This study employed the Polish version of CTQ (85). CTQ was administered once patients were clinically stabilized, within three months of admission.

### 2.3 Blood collection

Venous blood samples were obtained on the first post-admission morning (post-admission Day 1) between 7:00 and 9:00 a.m., after an overnight fast (≥8 h). For patients admitted overnight, sampling was performed on the first available fasting morning after admission to ensure standardized fasting conditions. Baseline clinical (PANSS, STAI) and cognitive (MoCA) assessments were performed as close as possible to the biomarker time point—typically on the same day as the fasting blood draw, or within 24 hours if required by clinical logistics.

Samples were collected into serum tubes, allowed to clot, and centrifuged; the serum was separated and used for subsequent analyses.

Serum concentrations of complement components C3 (C3c) and C4 (C4c) were measured by immunonephelometry using N Antiserum to Human C3c and N Antiserum to Human C4 (Siemens Healthineers), directed against the C3c and C4c fragments, respectively. Measurements were performed on an automated immunonephelometric system according to the manufacturer’s instructions. Calibration was performed using N Protein Standard SL, traceable to IFCC reference material. Samples were automatically diluted 1:20 prior to measurement.

The laboratory reference ranges were as follows: C3 (C3c), 0.9–1.8 g/L; C4 (C4c), 0.1–0.4 g/L. Internal quality control was performed using multi-level control materials (N/T PROT CONTROL SL/L, SL/M, and SL/H). Quality control was run after each calibration/reference-curve establishment, during each analytical run, and after opening a new antiserum vial. Patient results were released only after acceptable QC performance; out-of-range controls triggered repeat testing and, if confirmed, recalibration and corrective actions.

### 2.4 Statistical analysis

Descriptive statistics, expressed as the mean ± standard error (SE) and median (Q1, Q3), were employed to summarize the data. The distribution normality of continuous variables was evaluated using the Shapiro-Wilk W test. Both variables showed significant deviations from normality (C3: W = 0.915, p = 0.006; C4: W = 0.941, p = 0.042) (**Supplementary Figures 1**, **2**). Consequently, the assumption of normal distribution was not fulfilled, and nonparametric statistical methods were applied for further analyses.

For the longitudinal within-subject comparisons between baseline (T1) and week 12 (T2), we applied the Wilcoxon signed-rank test (paired, non-parametric), because the analyzed variables were not assumed to be normally distributed and measurements were obtained from the same individuals at two time points. Continuous variables are reported as median with interquartile range (Q1–Q3). The magnitude of change was quantified using an effect size r, calculated as r = Z/√N (where *Z* is the standardized Wilcoxon statistic and *N* is the number of paired observations). All tests were two-tailed, and statistical significance was set at p < 0.05.

Correlations between continuous variables were examined with Spearman’s rank correlation coefficient (r). Spearman’s rank correlation coefficient was used to assess the relationships between variables due to the characteristics of the data. Specifically, several measures, including PANSS, MoCA, STAI, and BMI changes (ΔT), did not necessarily meet the assumptions of normality and linearity required for Pearson’s correlation. Spearman’s test, being a non-parametric method, evaluates monotonic relationships based on ranked data and is robust to skewed distributions and outliers, making it suitable for analyzing clinical and psychometric scores in this study. We performed multiple-comparison correction using the Benjamini–Hochberg false discovery rate (FDR) procedure. For each set of correlation analyses, two-sided p-values were adjusted across all tests within the table, and results are reported as FDR-adjusted q-values. Statistical significance was defined as q < 0.05.

The Mann–Whitney U test was used to compare the medians and overall distributions of the analyzed continuous variables between the low vs. high C3 groups and between the low vs. high C4 groups; in both cases, groups were defined using a median split. The medians were calculated in the full study sample of 39 patients, yielding cut-offs of C3 = 1.4 g/L and C4 = 0.2 g/L, rather than laboratory reference-range boundaries (C3: 0.9–1.8 g/L; C4: 0.1–0.4 g/L). For both C3 and C4, subgroup sizes were high n = 17 and low n = 22. Cliff’s delta (δ) was used as a non-parametric effect size for between-group comparisons (Mann–Whitney U tests). It quantifies the degree of separation between two independent distributions and can be interpreted as the probability that a randomly selected observation from one group is larger than a randomly selected observation from the other group minus the reverse probability. Values range from −1 to +1, where 0 indicates no difference between groups, and the sign indicates the direction of the effect. The magnitude of δ was interpreted using conventional thresholds (|δ| ≈ 0.147 small, 0.33 medium, 0.474 large).

Aligned Rank Transform (ART) analyses were used to assess the effects of potential covariates on the study outcomes while avoiding the distributional assumptions of parametric ANOVA. ART is a nonparametric procedure that first aligns the data for each effect of interest, then rank-transforms the aligned responses, and finally fits standard linear models to the ranked data to obtain ANOVA-type F tests for main effects (and, if specified, interactions). Separate ART models were fitted for C3 at T1 and C4 at T1, including age and BMI at T1 as continuous covariates and gender, smoking status (yes/no), and treatment status (yes/no) as categorical factors.

All statistical analyses were conducted using Statistica v.13.3 software.

## 3 Results

### 3.1 Patients’ characteristics

The study included 39 patients undergoing psychotic decompensation, diagnosed with schizophrenia based on ICD-10 criteria, who were admitted to the inpatient wards of the Psychiatry Department and assessed at two time points: at the beginning of treatment and three months later. All 39 patients completed assessments at both time points (no loss to follow-up). The study focused on relatively young patients, with a mean age of 22.4 ± 1.2 years (SE). The duration of untreated psychosis ranged from 3 to 140 days. The mean length of hospitalization was 63.2 ± 5.2 days. Among the participants, 9 (23.1%) reported smoking, while 30 (76.9%) were non-smokers. All smokers reported consuming approximately one pack of cigarettes per day, with a smoking history ranging from 3 to 15 years. The mean body mass index (BMI) was 22.0 ± 0.7 kg/m² (SE), ranging from 14.5 to 38.4 kg/m², with a median of 21.05 kg/m². The change in BMI between T2 and T1 (ΔT = T2 − T1) indicated a modest overall increase in body mass across participants, with most individuals showing small gains within a limited range. Physical activity patterns differed across the cohort: most participants reported low activity (69.2%; mainly light daily movements or leisurely walking), some reported moderate activity (20.5%; brisk walking or cycling 2–4 times per week), and a smaller group reported high activity (10.3%; vigorous exercise at least 3 times per week or regular activity on ≥5 days per week). An overview of the general sociodemographic and clinical characteristics of the study sample is provided in **Table 1**.

**Table 1:** Sociodemographic and clinical characteristics of the study sample.

STUDY PARTICIPANTS	
N (%)	39 (100.0)
Sex	
Female N (%) Male N (%)	18 (46.2) 21 (53.8)
Age [years]	
Mean ± SE Range Median Q1, Q3	22.4 ± 1.2 15.0–38.0 18.0 17.0, 28.0
Tobacco smoking	
Yes N (%) No N (%)	9 (23.1) 30 (76.9)
BMI at time T1 [kg/m^2^]	
Mean ± SE Range Median Q1, Q3	22.0 ± 0.7 14.5–38.4 21.05 19.5, 24.2
BMI at time T2 [kg/m^2^]	
Mean ± SE Range Median Q1, Q3	22.8 ± 1.1 16.8 -39.1 22.2 20.6, 24.4
BMI ΔT (T2 − T1) [kg/m^2^]	
Mean ± SE Range Median Q1, Q3	1.0 ± 0.2 -2.1 - 4.4 1.1 0.5, 1.5
Physical Activity	
Low Medium High	27 (69.2) 8 (20.5) 4 (10.3)
Age of first episode of psychosis [years]	
Mean ± SE Range Median Q1, Q3	18.8 ± 1.6 12.0 – 29.0 17.5 16.0, 21.0
Duration of untreated psychosis [days]	
Mean ± SE Range Median Q1, Q3	24.3 ± 4.3 3.0 – 140.0 14.0 7.0, 30.0
Length of hospitalization [days]	
Mean ± SE Range Median Q1, Q3	63.2 ± 5.2 14.0 – 171.0 56.5 47.0, 79.0
Number of psychosis episodes	
Mean ± SE Range Median Q1, Q3	3.5 ± 0.6 1.0-15.0 1.0 1.0, 5.0
Duration of illness (years)	
Mean ± SE Range Median Q1, Q3	3.8 ± 0.9 0.1 – 21.0 0.3 0.2, 6.0
Treatment at admission	
Yes N (%) No N (%)	28 (71.8) 11 (28.2)

• Complete data were available for all 39 patients.

At admission, 11 patients (28.2%) were not receiving any antipsychotic medication. Among those treated, 26 patients (66.7%) received monotherapy and 2 patients (5.1%) combination therapy. The most commonly used antipsychotics at baseline were haloperidol (n = 16), olanzapine (n = 8), aripiprazole (n = 3), risperidone (n = 2), and quetiapine (n = 1). After conversion to chlorpromazine equivalents (CPZ) (86), the median antipsychotic dose at baseline was 100 mg. Throughout the treatment period, pharmacotherapy was optimized, and after 12 weeks all patients were receiving antipsychotic medication. At follow-up, the most frequently prescribed drugs were olanzapine (n = 21), quetiapine (n = 12), and aripiprazole (n = 12), followed by risperidone (n = 3), clozapine (n = 2), and haloperidol (n = 2). The median dose at week 12 was 400 mg in CPZ equivalents. The therapeutic doses administered were well tolerated by all patients at both assessment points.

### 3.2 Clinical evaluations

The results of the clinical assessment scales are shown in **Table 2**. The CTQ assessment was conducted once the patients had achieved clinical stabilization within three months of admission. PANSS, STAI, and MoCA evaluations were performed at two time points: at the beginning of hospitalization (T1) and after the 12-week treatment period (T2).

**Table 2:** Results of clinical assessment scales collected at two time points: initial hospitalization (T1) and post 12-week treatment (T2).

Assessment Tool	Mean ± SE	Range	Median	Q1; Q3
PANSS at Admission*				
PANSS-P_1	27.9 ± 1.1	15.0 – 38.0	29.0	22.0; 34.0
PANSS-N_1	25.7 ± 0.9	12.0-39.0	26.0	23.0; 29.0
PANSS-G_1	54.1 ± 1.7	25.0 – 70.0	54.0	46.0; 63.0
PANSS-Total_1	107.7 ± 3.2	69.0 – 141.0	114.5	89.0; 126.0
PANSS After 12 Weeks of Treatment*				
PANSS-P_2	12.1 ± 0.6	7.0 – 21.0	11.5	10.0; 15.0
PANSS-N_2	15.7 ± 0.9	7.0 – 31.0	14.5	11.0; 19.0
PANSS-G_2	31.2 ± 1.1	21.0 – 54.0	30.0	27.0; 34.0
PANSS-Total_2	57.5 ± 2.6	39.0 – 93.0	57.5	48.0; 68.0
PANSS ΔT (T2 − T1)*				
PANSS-P	-15.5 ± 0.8	-26.0 - -5.0	-15.5	-20.0; -11.0
PANSS-N	-10.0 ± 0.9	-19.0 – 1.0	-10.5	-13.0; -7.0
PANSS-G	-22.9 ± 1.6	-46.0 – 0.0	-24.5	-29.0; - 16.0
PANSS-Total	-48.4 ± 2.7	-88.0 - -20.0	-45.0	-61.0; -37.0
MoCA at Admission*				
MoCA-1	15.5 ± 0.6	9.0 – 22.0	15.0	13.0; 18.0
MoCA After 12 Weeks of Treatment*				
MoCA-2	20.2 ± 0.7	13.0 – 28.0	21.0	16.0; 24.0
MoCA ΔT (T2 − T1)				
MoCA	4.7 ± 0.3	-1.0 - 9.0	4.0	3.0; 6.0
STAI at Admission*				
STAI-T_1	58.0 ± 1.1	47.0 -70.0	60.0	51.0; 65.0
STAI-S_1	56.6 ± 1.1	45.0 – 75.0	56.5	49.0; 61.0
STAI After 12 Weeks of Treatment*				
STAI-T_2	50.5 ± 0.7	43.0 – 60.0	50.0	47.0; 54.0
STAI-S_2	48.6 ± 0.7	41.0 – 58.0	48.0	46.0; 52.0
STAI ΔT (T2 − T1)*				
STAI-T	-8.2 ± 0.7	-20.0 - 0.0	-8.0	-10.0; -5.0
STAI-S	-7.8 ± 0.7	-17.0 – -1.0	-7.0	-11.0; -4.0
CTQ*				
CTQ_EN	16.9 ± 0.7	8.0 – 24.0	17.0	14.0; 20.0
CTQ_EA	13.9 ± 0.7	6.0 – 23.0	13.5	10.0; 18.0
CTQ_PA	9.1 ± 0.6	5.0 - 19.0	8.0	6.0; 12.0
CTQ_PN	11.7 ± 0.7	5.0 – 21.0	10.0	8.0; 16.0
CTQ_SA	6.2 ± 0.5	5.0 – 20.0	5.0	5.0; 5.0
CTQ_Total	67.9 ± 2.7	39.0 – 96.0	65.0	55.0; 85.0

*Complete data were available for all 39 patients for PANSS, MoCA, and STAI at both T1 and T2; CTQ was assessed once (N = 39).

The difference between T2 and T1 (ΔT = T2 − T1) was calculated for each participant to quantify changes over time in PANSS, MoCA, and STAI scores. PANSS scores decreased across all subscales, indicating an overall reduction in symptom severity. MoCA scores increased between the two assessment points, suggesting improved global cognitive performance. STAI scores decreased at follow-up, indicating lower levels of both trait and state anxiety, with variability across individuals.

Across 39 paired observations, paired-change (T1 vs T2) analyses using the Wilcoxon signed-rank test indicated statistically significant changes from T1 to T2 in all outcomes (**Table 3**). Symptom severity measured with PANSS decreased markedly across positive, negative, general, and total scores (all p<0.001; large effects, r≈0.854–0.873). MoCA increased (median 15.0 to 21.0; p<0.001; r=0.86), and both trait and state anxiety (STAI-T, STAI-S) decreased (p<0.001; r≈0.85–0.86). PANSS, MoCA, and STAI exhibited consistently large effects, indicating that the observed pre–post differences were not only statistically reliable but also substantial in magnitude.

**Table 3:** Baseline-to–week 12 changes in PANSS, MoCA, and STAI (Wilcoxon signed-rank test).

Feature	N	Median value at time T1, Q1, Q3	Median value at time T2, Q1, Q3	T	Z	P value	Effect size r
PANSS-P	39	29.0, 22.0; 34.0	11.5, 10.0; 15.0	0.00	5.37	< 0.001	0.863
PANSS-N	39	26.0, 23.0; 29.0	14.0, 11.0; 19.0	1.00	5.29	< 0.001	0.857
PANSS-G	39	54.0, 46.0; 63.0	30.0, 27.0; 34.0	0.00	5.30	< 0.001	0.854
PANSS-Total	39	114.5, 89.0; 126.0	57.5, 48.0; 68.0	0.00	5.44	< 0.001	0.873
MoCA	39	15.0, 13.0; 18.0	21.0, 16.0; 24.0	1.00	5.36	< 0.001	0.861
STAI-T	39	60.0, 51.0; 65.0	50.0, 47.0; 54.0	0.00	5.30	< 0.001	0.857
STAI-S	39	56.5, 49.0; 61.0	48.0, 46.0; 52.0	0.00	5.37	< 0.001	0.869

### 3.3 C3 and C4 concentrations at admission (T1) and exploratory covariate analysis (ART; N = 39)

In the group of 39 patients, C3 and C4 concentrations were measured at admission (T1). At baseline, C3 had a mean of 1.4 ± 0.06 g/L (SE) and a median of 1.4 g/L, whereas C4 had a mean of 0.2 ± 0.01 g/L (SE) and a median of 0.2 g/L.

In an exploratory aligned rank transform (ART) analysis, none of the evaluated covariates (age, sex, smoking status, BMI at T1, treatment status at admission (yes/no), or chlorpromazine-equivalent dose at T1 and T2) showed a statistically significant main effect on C3 or C4 concentrations measured at baseline (T1) (all p > 0.05; **Table 4**).

**Table 4:** Exploratory aligned rank transform (ART) analysis: p-values for the effects of individual covariates on C3 and C4 concentrations at T1 time.

Variables	Effect of single covariates in exploratory aligned rank transform analysis p value						
	Age	Sex	Smoking Yes/no	BMI (T1)	Treatment admission (yes/no)	CPZ (T1)	CPZ (T2)
C3 at time T1	0.322	0.854	0.860	0.214	0.310	0.326	0.282
C4 at time T1	0.500	0.353	0.462	0.469	0.283	0.067	0.248

CPZ, chlorpromazine equivalents; T1, baseline at admission; T2, week 12 follow-up.

### 3.4 Correlation between the duration of untreated psychosis and length of hospitalization and baseline levels of C3

**Figure 1:**
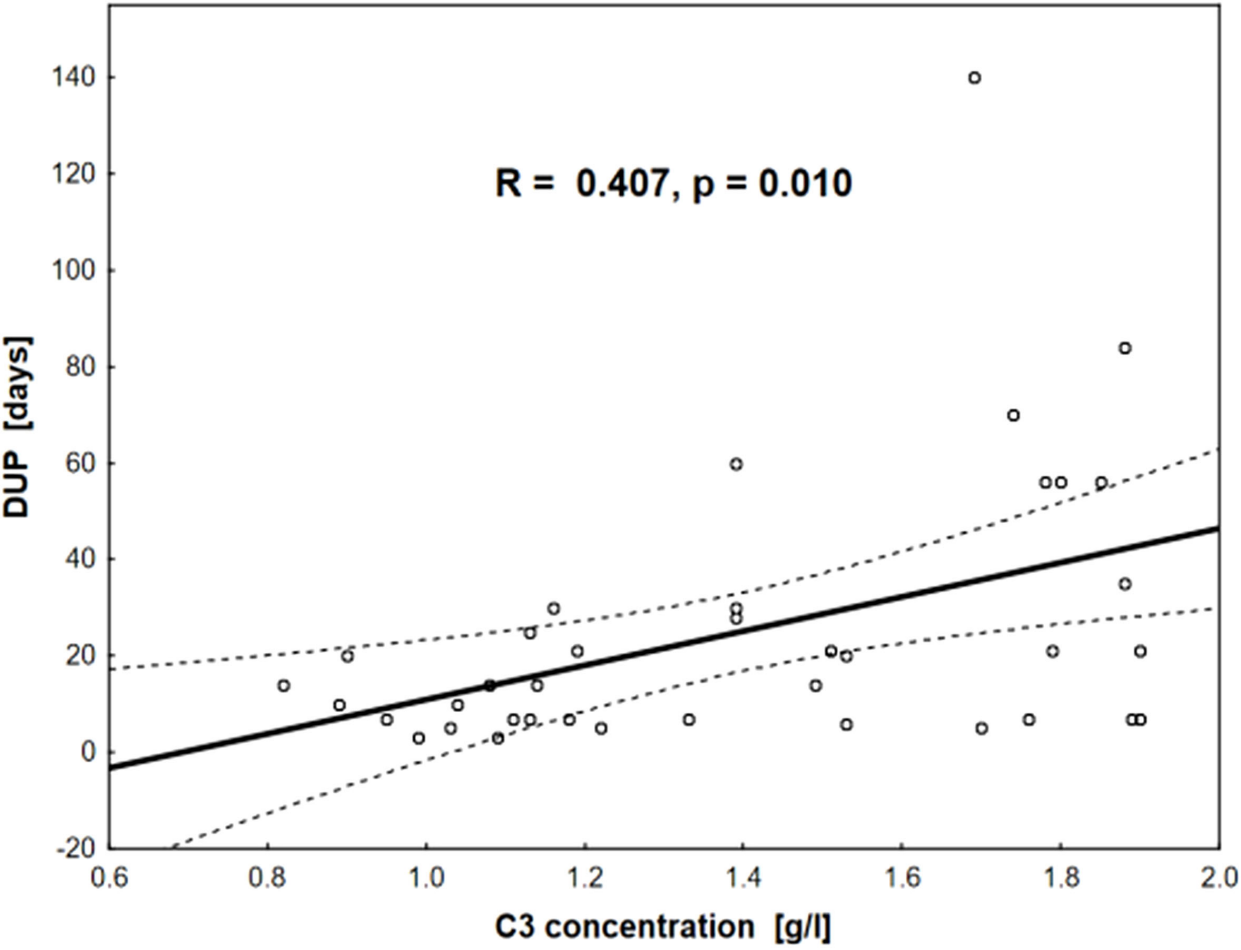
Correlation between baseline serum C3 concentration and duration of untreated psychosis analysed as continuous variables using spearman test.

**Figure 2:**
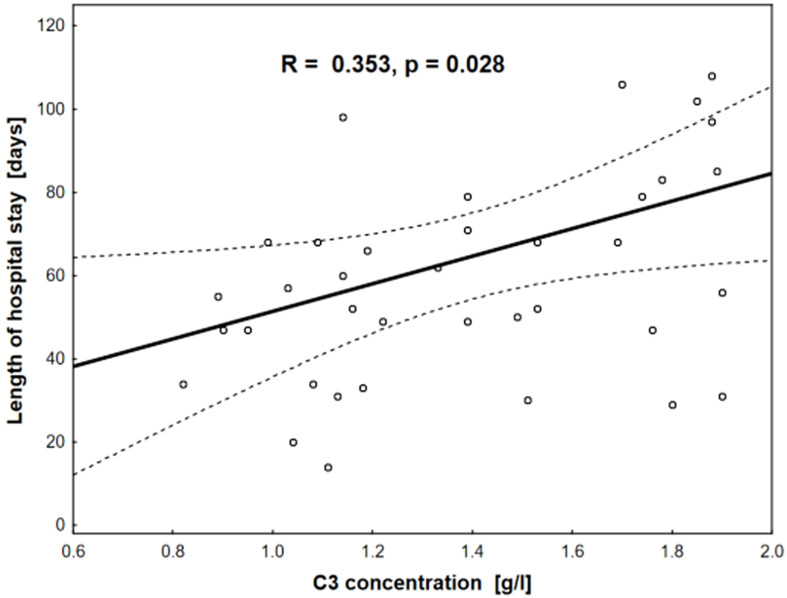
Correlation between baseline serum C3 concentration and length of hospitalization analysed as continuous variables using spearman test.

Baseline serum C3 concentrations exhibited positive correlations with both DUP and the length of hospitalization (r=0.407, p=0.010; r=0.353, p=0.028, respectively), as depicted in **Figure 1** and **2**. These associations were nominally significant (uncorrected). In contrast, C4 levels did not show significant correlations with either DUP or the length of hospitalization.

Furthermore, when patients were categorized into high and low C3 and C4 groups based on median values (1.4 g/L and 0.2 g/L, respectively), and the Mann-Whitney test was applied, no significant differences in DUP or the length of hospitalization were observed between patients with high vs. low C3 levels or high vs. low C4 levels (see **Supplementary Materials**, **Supplementary Tables 3**, **4**).

### 3.5 Correlation between the severity of psychotic symptoms measured by PANSS and baseline levels of C3 and C4

**Figure 3:**
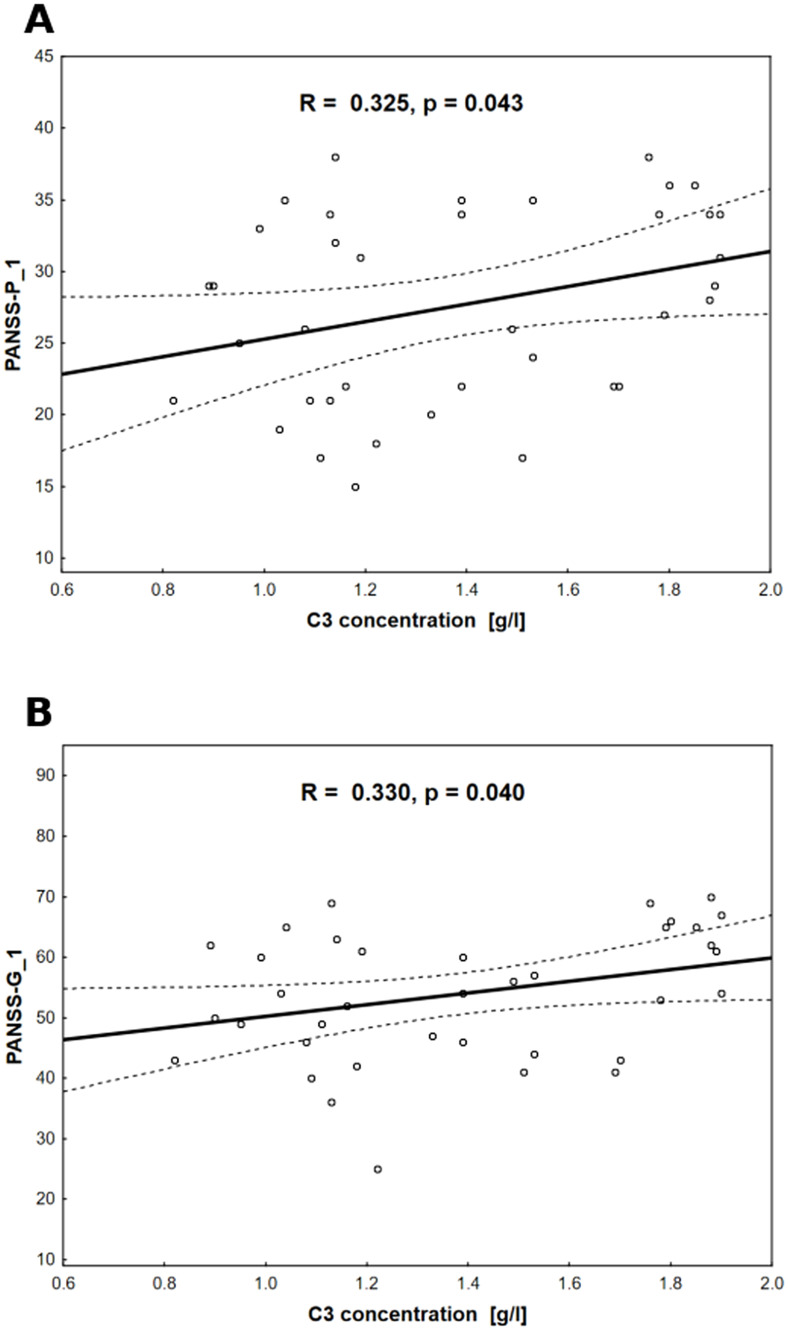
Correlation between baseline serum C3 concentrations and PANSS scores analysed as continuous variables using spearman test. **(A)** PANSS-P_1 subscale – Positive Symptoms at admission, **(B)** PANSS-G_1 subscale – General Psychopathology at admission.

**Figure 4:**
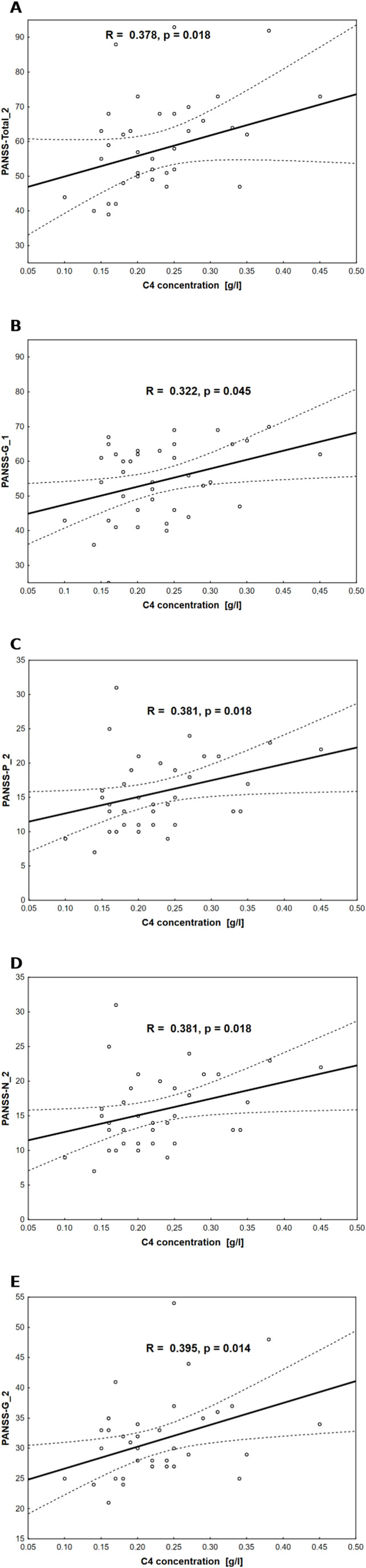
Correlation between baseline serum C4 concentrations and PANSS scores analysed as continuous variables using spearman test. **(A)** PANSS-Total_2 – Total score after 12 weeks, **(B)** PANSS-G_1 subscale – General Psychopathology at admission, **(C)** PANSS-P_2 subscale – Positive Symptoms after 12 weeks, **(D)** PANSS-N_2 subscale – Negative Symptoms after 12 weeks, **(E)** PANSS-G_2 subscale – General Psychopathology after 12 weeks.

The analysis revealed nominally significant positive correlations between baseline serum C3 concentrations and PANSS-P_1 and PANSS-G_1 (r=0.325, p=0.043; r=0.330, p=0.040, respectively) (**Figure 3**). Additionally, nominally significant positive correlations were observed between baseline serum C4 concentrations and PANSS scores measured at admission and after 12 weeks of hospitalization, including PANSS-G_1 (r=0.322, p=0.045), PANSS-P_2 (r=0.381, p=0.018), PANSS-N_2 (r=0.381, p=0.018), PANSS-G_2 (r=0.395, p=0.014), and PANSS-Total_2 (r=0.378, p=0.018) (**Figure 4**). Patients with higher C4 levels scored higher on PANSS-N_2, PANSS-G_2, and PANSS-Total_2 compared to those with lower C4 levels. No significant differences were found in PANSS subtests between patients with high and low C3 levels (see **Supplementary Materials**, **Supplementary Tables 3**, **4**).

### 3.6 Correlation between anxiety levels measured by STAI and baseline levels of C3 and C4

**Figure 5:**
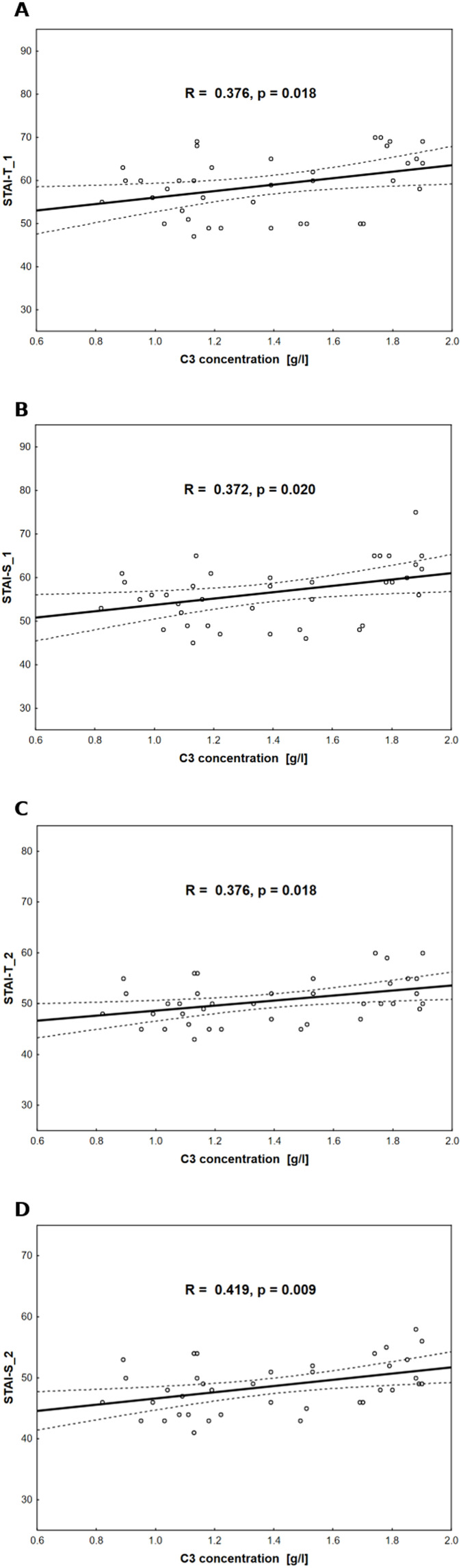
Correlation between baseline serum C3 concentrations and STAI scores analysed as continuous variables using spearman test. **(A)** STAI-T_1 – Trait Anxiety at admission, **(B)** STAI-S_1 – State Anxiety at admission, **(C)** STAI-T_2 – Trait Anxiety after 12 weeks, **(D)** STAI-S_2 – State Anxiety after 12 weeks.

**Figure 6:**
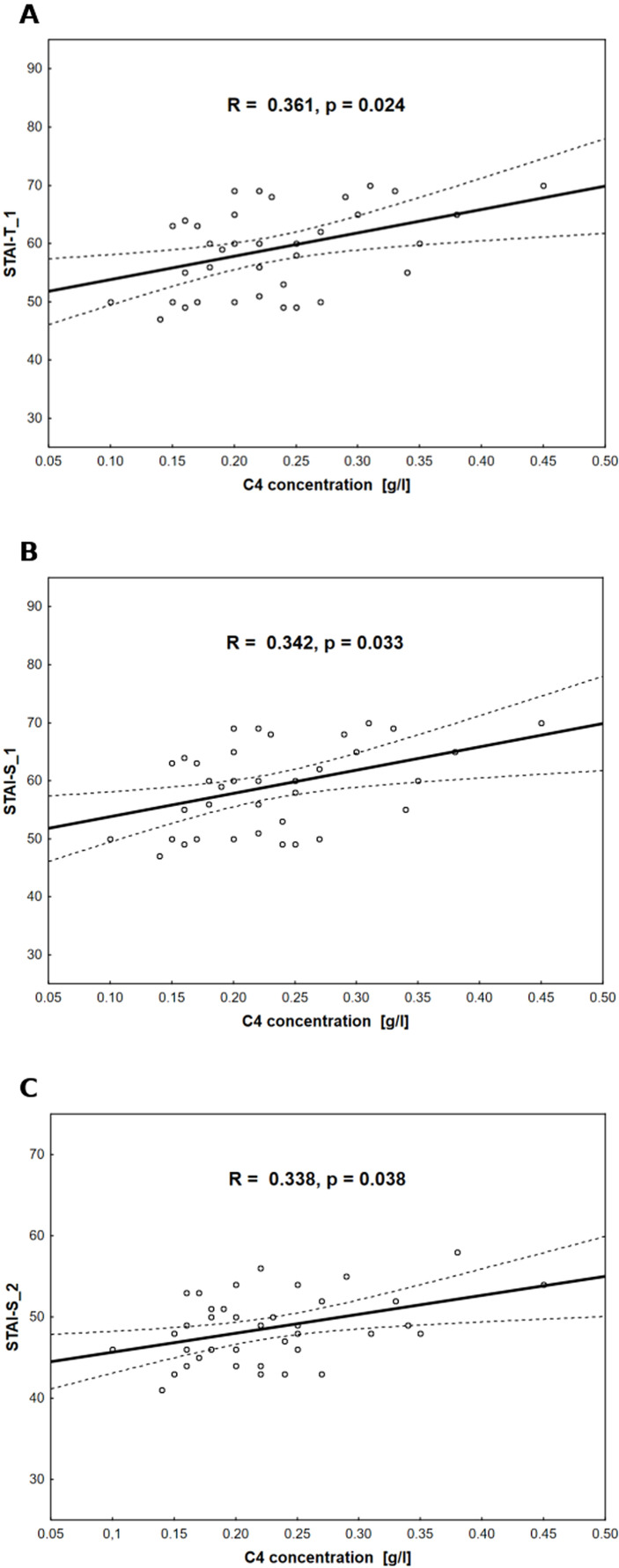
Correlation Between Baseline Serum C4 Concentrations and STAI Scores analysed as continuous variables using Spearman test. **(A)** STAI-T_1 – Trait Anxiety at admission, **(B)** STAI-S_1 **–** State Anxiety at admission, **(C)** STAI-S_2 – State Anxiety after 12 weeks.

Nominally significant positive correlations were observed between baseline serum C3 concentrations and STAI scores, including both trait and state anxiety, measured at the first and second assessment points: STAI-T_1 (r=0.376, p=0.018), STAI-S_1 (r=0.372, p=0.020), STAI-T_2 (r=0.376, p=0.018), and STAI-S_2 (r=0.419, p=0.009) (**Figure 5**). Nominally significant positive correlations were also found between baseline serum C4 concentrations and anxiety levels (both state and trait) at both time points: STAI-S_1 (r=0.342, p=0.033), STAI-T_1 (r=0.361, p=0.024), and STAI-S_2 (r=0.338, p=0.038) (**Figure 6**). Furthermore, the analysis revealed that individuals with high C3 levels scored higher on STAI-T_1, STAI-T_2, and STAI-S_2 compared to those with low C3 levels. No significant differences were found in STAI subtests between patients with high and low C4 levels using the Mann-Whitney test (see **Supplementary Materials**, **Supplementary Tables 3**, **4**).

### 3.7 Correlation between childhood trauma history assessed by CTQ and baseline levels of C3 and C4

**Figure 7:**
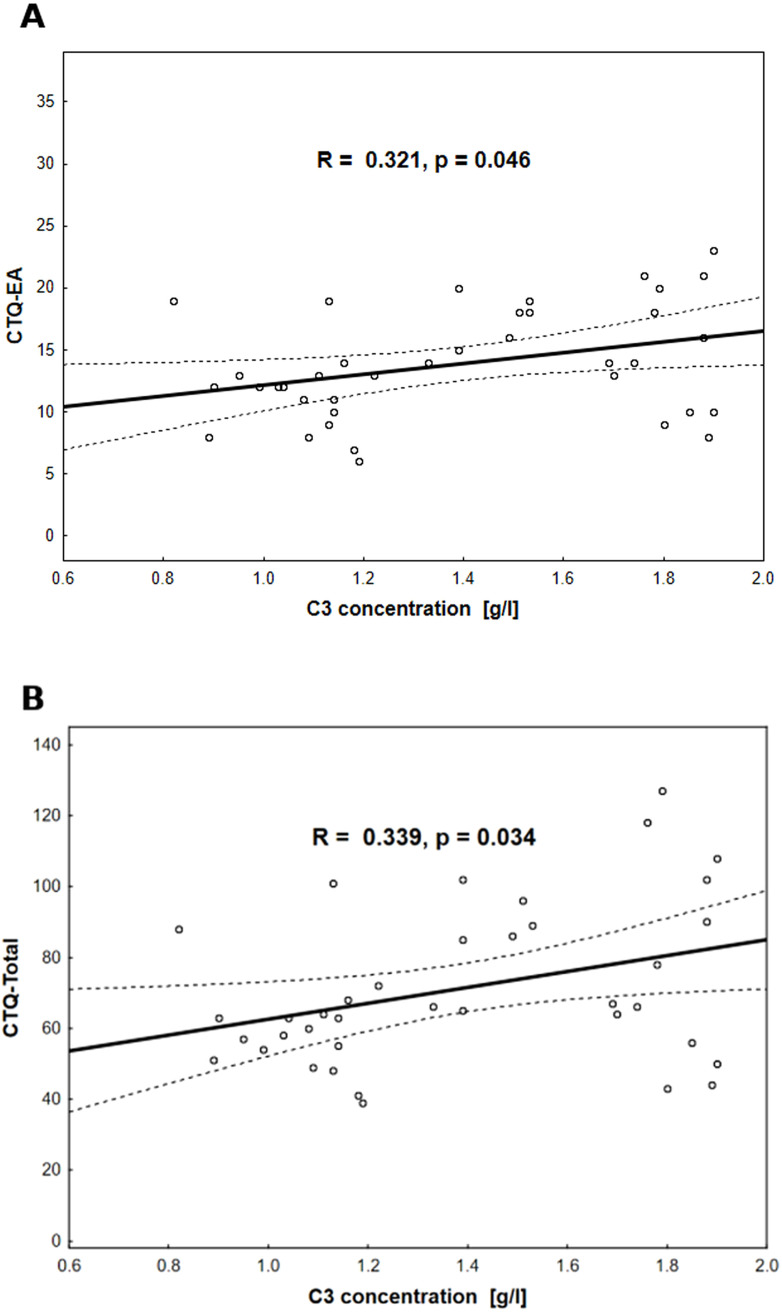
Correlation between serum C3 concentrations and **(A)** CTQ-EA subscale – Emotional Abuse, and **(B)** CTQ-Total, analysed as continuous variables using Spearman’s test.

**Figure 8:**
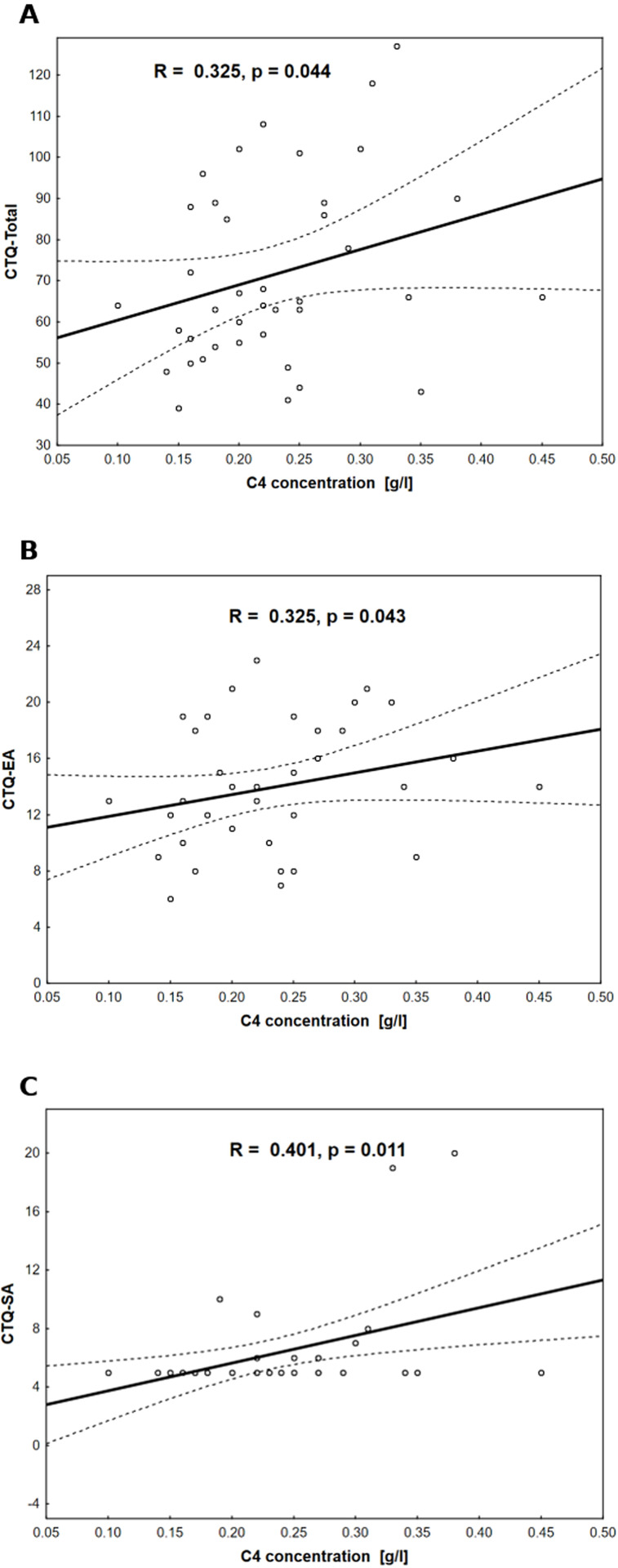
Correlation between serum C4 concentrations and **(A)** CTQ Total, **(B)** the CTQ-EA - Emotional Abuse, **(C)** CTQ-SA, Sexual Abuse, analysed as continuous variables using Spearman’s test.

Nominally significant positive correlations were observed between serum C3 concentration and CTQ scores, specifically CTQ-EA (r=0.321, p=0.046) and CTQ-Total (r=0.339, p=0.034) (**Figure 7**). Nominally significant positive correlations were found between serum C4 concentration and CTQ-EA (r=0.325, p=0.043), CTQ-SA (r=0.401, p=0.011) and CTQ-Total (r=0.325, p=0.044) (**Figure 8**). Furthermore, the analysis revealed that individuals with high C3 levels had higher scores on CTQ-EA, CTQ-PA, and CTQ-Total compared to those with low C3 levels. No significant differences were found in CTQ subtests between patients with high and low C4 levels, as assessed using the Mann-Whitney test (see **Supplementary Materials**, **Supplementary Tables 3**, **4**).

### 3.8 Correlation between cognitive functions assessed by MoCA and baseline levels of C3

**Figure 9:**
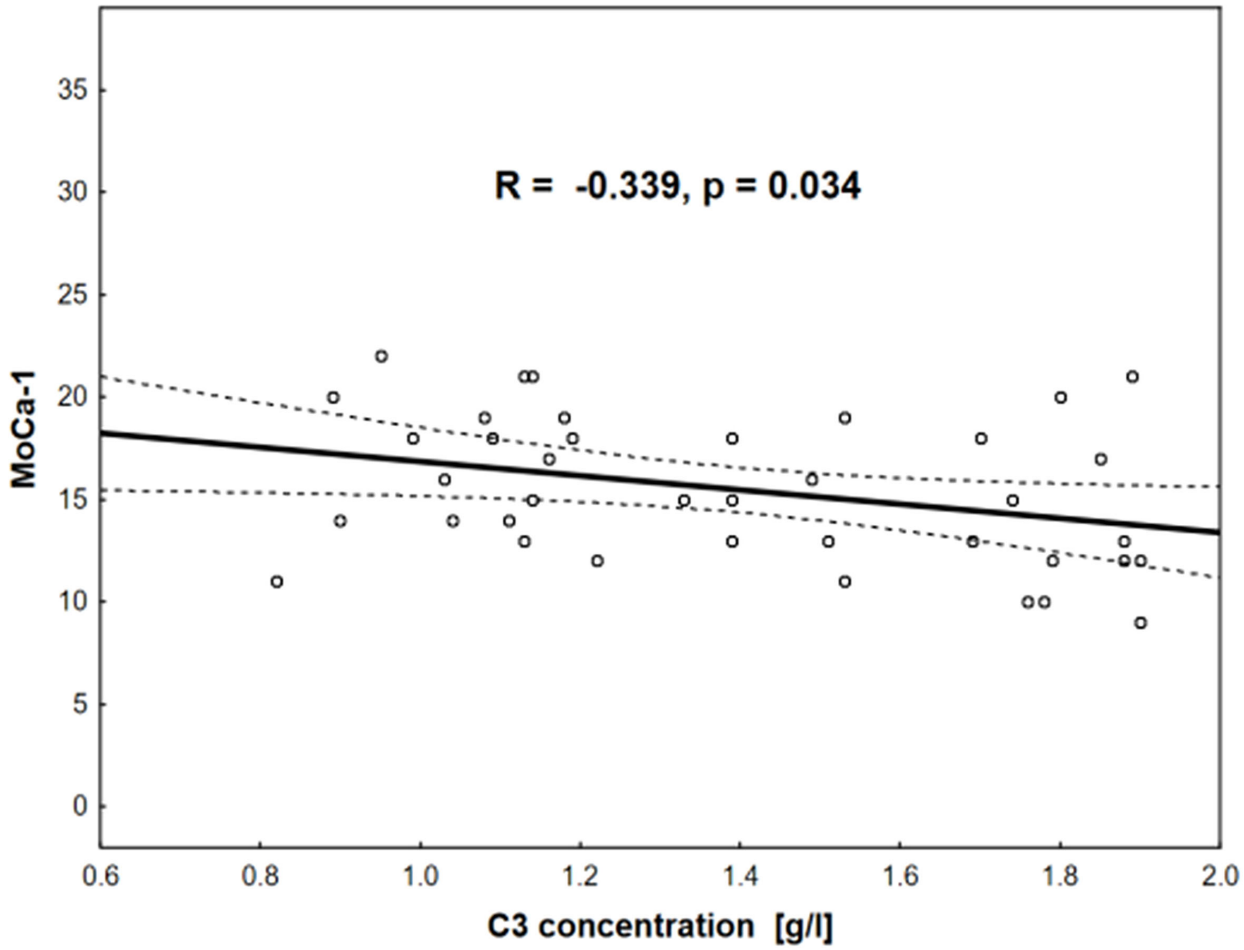
Correlation between baseline serum C3 Levels and MoCA scores analysed as continuous variables using spearman test. MoCA_1 – represents scores at admission.

The analysis revealed nominally significant negative correlations between baseline serum C3 levels and MoCA_1 (r=-0.339, p=0.034) at the first assessment point (**Figure 9**). Furthermore, patients with low C3 levels had higher MoCA_1 scores (at admission) and MoCA_2 scores (after 12 weeks) compared to those with high C3 levels. No significant differences were found in MoCA scores between patients with high and low C4 levels, as assessed using the Mann-Whitney test (see **Supplementary Materials**, **Supplementary Tables 3**, **4**). This suggests that lower baseline C3 levels may be associated with better cognitive performance over time.

No significant correlations were observed between the remaining parameters (demographic and clinical data) and complement C3 and C4 levels using both Spearman and Mann-Whitney tests (see **Supplementary Tables 1**–**4**).

No statistically significant differences in C3 or C4 levels were observed between patients receiving antipsychotic treatment at admission (yes) and those not receiving treatment (no) (both p > 0.05; **Supplementary Table 5**). Effect sizes were small to negligible (Cliff’s δ = 0.117–0.208), indicating at most a modest tendency toward higher C3 and C4 values in the treated group, with substantial overlap between distributions.

### 3.9 Multiple-comparisons correction (BH-FDR)

After correction for multiple comparisons using the Benjamini–Hochberg false discovery rate procedure, none of the associations with C3 or C4 concentrations reached statistical significance (all q-values > 0.05) (see **Supplementary Tables 1**, **2**).

## 4 Discussion

### 4.1 Complement C3 and duration of untreated psychosis in schizophrenia: a novel exploratory association

To the best of our knowledge, this is the first study to report a nominal, exploratory association between duration of untreated psychosis (DUP) and serum C3 levels in individuals with schizophrenia; however, this association did not survive BH-FDR correction for multiple comparisons. Peripheral complement abnormalities and markers of complement activation have been reported in schizophrenia and early psychosis across clinical subgroups. For example, higher serum C3/C4 has been associated with symptom severity and treatment resistance (56), elevated terminal complement activation (sTCC) has been observed in acutely ill, unmedicated patients (59), and baseline C4 has been reported to predict worse clinical outcome at 1-year follow-up in first-episode psychosis (92). In this context, our results generate the specific hypothesis that DUP may be linked to peripheral C3 variation. The higher C3 levels observed in our sample, a key component of the immune response, may reflect ongoing systemic inflammatory activity and, potentially, neuroimmune mechanisms relevant to schizophrenia. Such an increase in C3 might represent a response to tissue injury or contribute to pathological processes implicated in schizophrenia, including synaptic and neuroinflammatory pathways.

To contextualize the potential consequences of prolonged untreated psychosis, we previously examined DUP in relation to brain metabolic measures in the same cohort of individuals with schizophrenia. In that earlier analysis, we documented an inverse relationship between DUP and glucose concentration in the right frontal lobe, which may reflect reduced neuronal activity or altered energy utilization in this region (87). Additionally, we observed a positive correlation between DUP and choline levels in the anterior cingulate cortex (ACC), potentially indicating changes in membrane turnover or myelin-related processes (87).

By integrating these findings, it appears that schizophrenia, especially in its untreated form, is associated with a series of neurobiological alterations. These include disruptions in glucose metabolism, cell membrane integrity, and immune function, emphasizing the interconnected nature of these processes. This interplay among metabolic and neuroinflammatory processes further underscores the complex pathophysiology of schizophrenia and reinforces the urgent need for early and effective treatment interventions.

These observations support a promising, testable model in which a longer duration of untreated psychosis may be accompanied by both peripheral immune dysregulation and brain metabolic alterations. However, complement markers and proton magnetic resonance spectroscopy (^1H-MRS)–derived brain metabolite measures were not analysed jointly in the present study; therefore, this integrative interpretation remains hypothesis-generating and should be evaluated in future multimodal analyses.

### 4.2 Complement C3 and hospital length of stay in schizophrenia: an exploratory association

To the best of our knowledge, this is the first study to report a nominal association between higher baseline serum complement C3 levels and longer duration of hospitalization in schizophrenia. Furthermore, in exploratory analyses we observed that both C3 and C4 levels correlate with schizophrenia symptoms, as assessed using PANSS, at hospital admission and after three months of hospitalization (see Section 4.3). These nominal results suggest a broader link between immune dysregulation and the clinical severity of schizophrenia.

If we consider the length of hospital stay as a marker of recovery, our findings align with previous research demonstrating an association between elevated immune biomarker levels and poor treatment response. For instance, higher levels of interleukin-8 (IL-8) have been linked to poor antipsychotic response in patients with established schizophrenia (88). Similarly, increased levels of IL-6 and interferon-γ (IFN-γ) have been associated with poor treatment outcomes in patients with first-episode psychosis (89). Additionally, patients with chronic schizophrenia who exhibited higher baseline serum concentrations of IL-2 or IL-8 showed less improvement following treatment (90). C-reactive protein (CRP) predicts worse clinical outcome at 1-year follow-up in patients with first-episode psychosis (91). High baseline levels of complement component 4 predict worse clinical outcome at 1-year follow-up in first-episode psychosis (92). Consistent with our findings, elevated subclinical systemic inflammation, reflected by the neutrophil-to-lymphocyte ratio (NLR), was associated with longer hospitalization in patients with psychosis spectrum disorders, further supporting the link between immune dysregulation and recovery trajectories (93).

A potential explanation for our findings is that individuals with schizophrenia who present with higher C3 levels may have impaired recovery processes, resulting in prolonged hospitalization.

Neuroimaging studies further support this link, showing that increased inflammation has been implicated in treatment resistance in patients with schizophrenia by affecting brain structure and function. Specifically, reduced hippocampal volume and decreased cortical thickness have been identified as key neuroimaging markers associated with heightened peripheral inflammation (94).

An alternative hypothesis is that individuals with higher C3 levels may experience more severe psychotic episodes, requiring a longer treatment duration to achieve clinical stabilization and meet criteria for hospital discharge. This hypothesis is consistent with our observed nominal association between higher complement protein levels and the severity of psychosis symptoms. Given that complement components play a crucial role in synaptic pruning and neuroinflammatory processes and may contribute to blood–brain barrier disruption—processes that have been implicated in the pathophysiology of schizophrenia—it seems reasonable that relatively higher levels within our cohort may contribute to exacerbated psychotic symptoms.

In this context, complement components could be explored as candidate biomarkers of disease severity and treatment response, in line with prior reports involving IL-6, CRP, and IL-8. Future studies, ideally well-powered and mechanistically informed, should examine whether targeting complement pathways—via anti-inflammatory interventions or complement inhibitors—may influence treatment response and hospitalization duration in schizophrenia.

### 4.3 Complement and symptom severity in schizophrenia

In the present study, we observed that peripheral complement components were nominally associated with the severity of psychotic symptoms. At hospital admission, higher baseline C3 levels nominally correlated with greater symptom severity in the PANSS Positive and General Psychopathology subscales, whereas baseline C4 levels nominally correlated with General Psychopathology. Importantly, baseline C4 also showed nominal associations with symptom severity after 12 weeks of inpatient treatment, correlating with PANSS Positive, Negative, General Psychopathology, and Total scores at follow-up. Collectively, these results indicate that, in our cohort, higher baseline complement levels—particularly C4—were nominally associated both with symptom burden at admission and with symptom severity at the 12-week follow-up.

Recent studies provide mixed but informative context for these observations. In chronic schizophrenia, Göker et al. reported significantly higher serum C3 and C4 levels in treatment-resistant patients than in patients in remission and healthy controls. Complement levels were positively associated with symptom severity, and higher C4 increased the odds of treatment resistance in multivariable analyses (56). In first-episode, drug-naïve schizophrenia, Cao et al. similarly observed elevated serum C3 and C4 compared with controls. In symptom analyses, C3 showed a positive association with positive symptom severity, whereas C4 did not show significant correlations with symptom ratings beyond the case–control difference (57). These findings are broadly compatible with our data, supporting the possibility that elevations in peripheral complement factors—particularly C3—may accompany greater symptom severity in at least some illness stages or clinical subgroups.

In contrast, other work suggests that peripheral complement alterations may relate more strongly to cognitive or brain structural correlates than to PANSS-rated symptom severity. Yu et al. reported higher plasma C4 (but not C3) in drug-naïve first-episode schizophrenia; within patients, higher C3 was associated with poorer delayed logical memory, whereas higher C4 was associated with cortical thinning, and no significant associations between C3/C4 levels and PANSS scores were identified (58). Similarly, Savukoski et al. reported higher plasma levels of the soluble terminal complement complex (sTCC/sC5b-9) in acutely ill, unmedicated schizophrenia patients compared with matched controls; however, sTCC was not associated with PANSS symptom severity and did not change significantly after 6 weeks of treatment. In a serum sub-sample, C4 likewise showed no case–control difference and no associations with symptom ratings (59). Taken together, these mixed findings suggest that peripheral complement alterations may, in some settings, relate more strongly to cognitive/structural brain correlates or represent a trait-like marker of immune activation rather than a direct index of symptom intensity.

Overall, our data add to the literature suggesting that peripheral complement dysregulation may be linked to symptom burden in schizophrenia, with baseline C4 showing the most consistent nominal associations with symptom severity over short-term inpatient treatment. Future studies should examine whether a broader complement panel, covering upstream components (e.g., C3/C4) and downstream activation markers (e.g., sTCC), is reproducibly associated with clinically meaningful phenotypes in schizophrenia, including potential immune-related subgroups and longitudinal outcomes.

### 4.4 Comparison with recent complement–schizophrenia studies

To place our findings in the context of complement–schizophrenia research, we compared our study with four key recent publications (56–59). A structured overview is provided in **Supplementary Table 6**.

#### 4.4.1 Design differences

Across studies, designs vary in illness stage and medication exposure, as well as in the comparison framework and follow-up. They also differ in biomarker scope, blood-draw timing, laboratory procedures, and outcome definitions (e.g., treatment resistance/remission status, diagnostic performance, and MRI–cognition associations versus our focus on DUP, length of hospitalization, anxiety and childhood trauma, and symptom and cognitive measures), which likely contributes to heterogeneity in findings (56–59).

#### 4.4.2 Similarities/differences in findings across studies

Taken together peripheral complement abnormalities have been reported in schizophrenia across clinical contexts, including early psychosis, chronic illness, and acute presentations (56–59). Several reports—including ours—link higher C3/C4 to clinically relevant phenotypes: Göker et al. (2023) reported higher C3/C4 in treatment-resistant patients (vs remission and controls) and associations with symptom severity (56), and Cao et al. (2023) found elevated C3/C4 in first-episode schizophrenia, with C3 associated with positive symptom severity (57). Consistent with this, in our cohort higher baseline C3/C4 were associated with greater symptom severity; baseline C4 was also associated with symptom severity at follow-up, and baseline C3 with longer DUP and a longer length of hospitalization.

In contrast, other studies highlight different complement signals and phenotypic anchors: Yu et al. (2023) primarily linked complement variability to neurobiological/cognitive measures (cortical thinning and logical memory) rather than symptom ratings (58), while Savukoski et al. (2024) highlighted downstream/terminal pathway activity: plasma sTCC was elevated in patients versus controls, whereas C4 did not differ; moreover, sTCC and C4 showed no significant change after 6 weeks of antipsychotic treatment and were not associated with PANSS-rated symptom severity (59).

#### 4.4.3 Potential explanations for discrepancies

Although the reviewed studies consistently suggest that peripheral complement alterations can be detected in schizophrenia, the direction and clinical correlates of these signals vary across reports. Discrepancies among recent complement–schizophrenia studies likely reflect the fact that each investigation samples a different point on the illness × treatment continuum. Cohorts include drug-naïve first-episode schizophrenia (minimising medication confounding and capturing “early” biology) as well as chronic patients—acutely ill, treatment-resistant, or in remission—in whom repeated episodes, long-term antipsychotic exposure, and co-occurring metabolic and inflammatory burden may reshape baseline complement tone. In parallel, designs differ in comparison framework and follow-up: most studies use cross sectional case–control contrasts (optimised to detect between group shifts), whereas our within-cohort design without healthy controls emphasises how baseline serum C3/C4 at admission nominally relates to clinically meaningful variation such as DUP and length of hospitalization, and nominally correlates with PANSS severity at two time points (admission and 12 weeks).

Methodological differences may also explain why results vary: studies used different sample types (serum vs EDTA plasma), different laboratory methods (nephelometry vs ELISA/multiplex), and often targeted different parts of the cascade—either upstream components (C3/C4) or activation products (sTCC). They also differed in the timing and processing of blood draws, which can add variability to complement measures—for example, morning fasting collections in some cohorts versus late-afternoon sampling in Yu et al. (2023; 4:00–4:30 p.m.) (58). As a result, the reviewed studies may be capturing slightly different “complement signals, “ limiting direct comparability across reports. Upstream C3/C4 and downstream activation markers likely capture different layers of the same system. C3/C4 primarily index complement availability and often relate to overall clinical burden (56, 57)—including in our cohort, where baseline C3/C4 were associated with PANSS severity. In contrast, Savukoski et al. found elevated sTCC (with higher CRP) but no differences in C4 and no associations with PANSS severity or treatment-related change, consistent with a more trait-like immune activation signal (59).

Overall, the emerging picture is that peripheral complement dysregulation is a reproducible feature of schizophrenia, but it is not expressed uniformly across cohorts. Which signal emerges as “most informative” appears to depend on who is studied (illness stage, symptom state, medication exposure), what is measured (upstream component levels such as C3/C4 versus downstream/terminal activation markers), and the chosen clinical anchors (symptoms versus cognition/brain structure). Taken together, these comparisons suggest that complement markers are most likely to be clinically useful when interpreted in context and, ideally, assessed within broader, standardized panels and well-phenotyped longitudinal designs that can disentangle state- from trait-like immune signals.

### 4.5 Complement and anxiety in schizophrenia: a novel exploratory association

To the best of our knowledge, this study is the first to report a nominal association between higher levels of anxiety (both state and trait), measured at baseline and after three months, and higher concentrations of complement C3 and C4 within our cohort; however, these associations did not survive BH-FDR correction for multiple comparisons. Our findings are broadly in line with previous evidence linking complement activity to anxiety-related outcomes. In animal models, heightened prenatal complement activity has been shown to result in anxiety-related phenotypes. Specifically, foetuses that exhibited increased C3 levels in their brains demonstrated anxiety-related behaviour postnatally (95). Aged C3-deficient mice exhibited lower levels of anxiety (96). Research by Yilmaz et al. (47) suggests that enhanced synaptic elimination mediated by C4A in a mouse model leads to the development of abnormal brain circuits and anxiety-related phenotypes. Moreover, a correlation between C3 levels and the severity of anxiety in patients with neurological disorders has been identified (97), and it has been observed that complement inhibitors can reduce anxiety associated with neurodegeneration (98).

Alterations in the expression of C3 and C4 may influence brain function and structure by promoting neurotoxic inflammation, excessive synaptic pruning, and synaptic loss (99, 100). These changes may disrupt neuroplasticity, affecting the brain’s ability to adapt to new environments and stressors, potentially exacerbating anxiety symptoms. In particular, higher C3 levels may contribute to atypical synaptic pruning, disrupting neural networks essential for emotional regulation and contributing to anxiety disorders. Inflammation resulting from altered C3 and C4 expression may also interfere with neurotransmitter regulation, further heightening anxiety symptoms.

Moreover, heightened anxiety and stress can also lead to changes in complement protein levels. For instance, exposure to brief but acute naturalistic stress has been shown to increase plasma C3 levels (101), while C3a levels were observed to rise immediately following an acute psychological stress task (102). Similarly, both C3 and C4 levels were found to be higher in a highly stressed group of students during academic exams, with levels returning to baseline several weeks later (103).

In the context of schizophrenia, it is hypothesised that there might be a feedback loop between complement components and anxiety. Relatively higher C3 and C4 levels could potentially exacerbate anxiety symptoms through mechanisms such as neuroinflammation, aberrant synaptic pruning, and disrupted neuroplasticity. Conversely, the heightened anxiety and stress commonly observed in individuals with schizophrenia might contribute to higher C3 levels. This hypothesis proposes a complex, bidirectional relationship in which each factor may intensify the other, potentially creating a self-reinforcing cycle.

### 4.6 Complement and childhood trauma in schizophrenia: a novel, exploratory association in the context of neuroinflammation

To the best of our knowledge, we are the first to report nominal, exploratory associations between childhood trauma experiences and higher peripheral C3 and C4 levels in patients with schizophrenia. Previous studies have shown that early-life trauma is related to C3 alterations in adulthood in animal models (104), and childhood traumatic experiences have been linked to C3 and C1q levels in healthy populations (105).

In our study, higher complement components may be interpreted as part of the inflammatory response. Previous research has demonstrated associations between childhood trauma and elevated inflammatory markers in adulthood, such as CRP, IL-6, and TNF-α (61).

However, given that the complement system maintains a strong functional association with inflammatory cytokines and interacts with them in a bidirectional manner (106), the higher complement activity observed in our study may itself be primarily responsible for the heightened inflammatory tone reported in previous studies.

Our findings are consistent with the hypothesis that activation of the complement system may be triggered by early-life trauma in individuals with schizophrenia. This activation plays a crucial role in neurodevelopmental processes.

For instance, the complement pathway is essential for neuronal migration (34), and its impairment has been linked to developmental disorders, including schizophrenia (107). This implies that trauma-induced dysregulation of the complement system may contribute to the developmental abnormalities associated with schizophrenia. Furthermore, the role of the complement system extends to the proliferation and differentiation of neural progenitor cells (NPCs) and neuroblasts (108), as well as affecting the morphology of immature neurons, especially in terms of dendritic arborization (109). These processes are vital for proper brain development, and their alteration due to trauma-induced complement activation could lead to the neuropathological changes observed in schizophrenia, such as reduced dendritic spine density (110). Additionally, the involvement of complement proteins in activity-dependent synaptic pruning during development (111) suggests that dysregulation in this system, as a result of trauma, could lead to long-term deficits in functional connectivity. This is further supported by findings of reduced synaptic densities in cortical regions in schizophrenia, which may reflect excessive synaptic elimination (112).

In summary, the higher levels of C3 and C4 in schizophrenia patients with a history of developmental trauma may be interpreted as an indication of the intricate link between traumatic experiences in early life, activation of the complement system, and the resulting neurobiological changes that contribute to the onset and progression of schizophrenia. However, it should be noted that although childhood trauma in our study was assessed using a reliable and validated questionnaire, the retrospective nature of the Childhood Trauma Questionnaire implies that the possibility of bias in self-reported experiences due to recall distortion or reinterpretation cannot be ruled out. Therefore, there may be a bidirectional relationship between complement molecule levels and the reported history of childhood trauma. On one hand, childhood trauma may contribute to higher levels of key complement proteins; on the other hand, individuals with higher complement components may be more likely to subjectively report traumatic experiences.

We also observed a nominal association between higher complement C3 and C4 concentrations and higher anxiety levels (see Section 4.5). Patients with higher complement proteins may have greater neurobiological susceptibility to stress, which could influence the way they interpret past experiences. Prolonged, chronic complement activation may contribute to a persistent inflammatory state, increase hypothalamic-pituitary-adrenal (HPA) axis activity, or disrupt neurodevelopmental processes. Individuals with higher C3/C4 levels may be more sensitive to stressors, leading to heightened anxiety and trauma responses. This, in turn, could affect the way past experiences are perceived and interpreted, potentially resulting in a higher frequency of trauma reporting among individuals with higher C3/C4 levels.

### 4.7 Complement C3 and cognitive performance in schizophrenia

Our findings show a nominal, exploratory association between complement activity and cognitive functioning in patients with schizophrenia. Specifically, lower levels of complement component C3 were nominally associated with higher MoCA scores obtained upon hospital admission. This observation is consistent with prior evidence linking peripheral complement alterations to cognitive and brain structural correlates in schizophrenia. Yu et al. (2023) reported that higher plasma C3 levels were associated with poorer delayed logical memory recall in drug-naïve patients with first-episode schizophrenia, while elevations in other peripheral complement components were linked to cortical thinning (58).

Our results regarding the nominal association between complement and cognitive function are plausible given the multiple roles of the complement system in the CNS. It is essential for nearly all aspects of the development of the CNS, including neurogenesis (113, 114), neuronal migration (34), synaptic refinement (115), and the regulation of neuronal plasticity (116). Additionally, it plays a crucial role in maintaining and restoring homeostasis in the CNS by protecting against infection and inflammation, clearing apoptotic cells, debris, and plaques, and supporting neural protection, repair, and regeneration (117, 118).

However, in addition to its neuroprotective effects, complement activation may also exert neurotoxic and deleterious effects in the CNS. The complement system has been implicated in age-related synaptic loss and cognitive decline (96, 119) and in immune-mediated mechanisms that can contribute to CNS injury and functional impairment across a range of neurological disorders, including multiple sclerosis, neuromyelitis optica, neurotrauma, stroke, amyotrophic lateral sclerosis, Alzheimer’s disease, Parkinson’s disease, and Huntington’s disease (32, 120).

Our results are consistent with observations from preclinical models suggesting that C3 deficiency may protect against cognitive impairment and synaptic and neuronal loss. It was demonstrated that C3-deficiency spared age-dependent synaptic and neuronal loss in a region-specific manner and protected against cognitive impairment in normal aging of mice (96). Moreover, in an Alzheimer’s disease model, C3 deficiency was protective against age- and plaque-related synapse and neuron loss, decreased glial reactivity, reduced the production of some pro-inflammatory cytokines, and spared cognitive decline despite an increased plaque burden in the mouse brain (121). Pharmacological inhibition of C3 activity was sufficient to prevent early dendritic synaptic loss within the dentate gyrus, mediated by microglial phagocytosis, and memory impairment in mice with experimental autoimmune encephalomyelitis (122). C3 also plays a role in regulating the number and function of glutamatergic synapses in the hippocampus and exerts negative effects on hippocampus-dependent cognitive performance in mice (119). These data highlight the central role of C3 in early hippocampal synaptic vulnerability and memory impairment (119).

Moreover, excessive activation of the complement system can exacerbate neuroinflammation by stimulating microglia, promoting the release of pro-inflammatory cytokines, and increasing the production of reactive oxygen species (123). In schizophrenia, C3 overactivation may be part of a broader neuroinflammatory response, and increased levels of inflammatory markers, particularly C-reactive protein (CRP) and tumour necrosis factor-alpha (TNF-α), have also been linked to cognitive dysfunction in schizophrenia (124). These findings raise the possibility that targeting neuroinflammation, including complement regulation, may be relevant to cognitive deficits in schizophrenia; however, this remains speculative and requires confirmation in larger, controlled studies, including interventional designs.

## 5 Strengths

We employed a broad and clinically meaningful assessment strategy by combining measures of anxiety (STAI), cognitive performance (MoCA), and childhood trauma (CTQ) with standard symptom ratings (PANSS). These domains are rarely examined together in relation to peripheral complement components (C3/C4) in schizophrenia, making their integrated evaluation a novel feature of our study.

Our analyses yielded several novel, exploratory (hypothesis-generating) associations, including nominal links between C3 and both the duration of untreated psychosis and length of hospitalization, as well as nominal associations between complement levels and anxiety severity and childhood trauma. To the best of our knowledge, these relationships have not been previously reported for peripheral complement markers in schizophrenia, underscoring the potential value of our results for guiding future research in this emerging area.

A further strength is the two-time-point clinical evaluation (at admission and after 12 weeks of active inpatient treatment), which captured symptom, anxiety, and cognitive changes during an understudied yet clinically dynamic phase of schizophrenia. This design allowed us to assess whether baseline peripheral C3/C4 levels relate not only to cross-sectional psychopathology but also to short-term clinical trajectories.

Importantly, our results align with recent evidence, supporting their interpretability in a broader context. The association between C3 and cognition is consistent with Yu et al. (2023) (58), while the correlations between C3/C4 and symptom severity resemble patterns reported by Göker et al. (2023) (56) and Cao et al. (2023) (57). Overall, our findings are in line with literature suggesting stage-dependent complement dysregulation in schizophrenia (56).

Finally, the sample size (n = 39) is reasonable for hypothesis-generating correlational research and supports the stability of the observed associations.

## 6 Limitations

The study has several limitations that should be considered. First, the present work was designed as exploratory and hypothesis-generating and was conducted in a relatively small cohort (n = 39). In line with this exploratory aim, we examined a broad set of clinically meaningful associations between baseline peripheral complement markers (C3/C4) and multiple symptom, anxiety, trauma, cognitive, and illness-course measures. We report effect sizes (Spearman’s r) alongside nominal (uncorrected) p-values and additionally provide Benjamini–Hochberg false discovery rate (FDR)–adjusted q-values to account for multiple testing. Importantly, after FDR correction, the associations with C3 or C4 did not remain statistically significant (all q-values > 0.05), indicating that the nominally significant findings should be interpreted cautiously. Given the number of tests and the limited sample size, the findings are preliminary and require replication in larger, independent cohorts with pre-specified primary hypotheses and an appropriate multiplicity-control strategy.

Second, the lack of a matched healthy control group limits the generalizability of the findings and prevents direct comparisons with non-schizophrenia individuals. In particular, the absence of controls precludes conclusions about absolute elevation of C3/C4. Most values fell within laboratory reference ranges; therefore, our findings should be interpreted as within-cohort associations rather than evidence of complement levels above the reference range.

Third, complement components were measured only once—at admission—providing a cross-sectional snapshot that precludes assessment of temporal dynamics and limits inference about causality or whether complement markers change with treatment or symptom fluctuations. Consistent with this, although baseline C3/C4 levels were associated with clinically meaningful outcomes (e.g., DUP and length of hospitalization) and with symptom severity, anxiety burden, and cognitive performance at baseline and/or follow-up, we did not observe consistent associations between baseline complement levels and simple pre–post change scores (ΔT2–T1) on clinical scales. This may reflect a methodological limitation of using raw change scores, which are inherently anchored to baseline values and may not provide an independent measure of clinical improvement (e.g., participants with higher baseline scores have greater potential for reduction). Therefore, the absence of biomarker–Δ associations should be interpreted cautiously and does not negate the observed cross-sectional and outcome-related associations.

Fourth, the study relied on peripheral C3 and C4 measurements, which may not fully reflect complement activity in the brain due to the blood-brain barrier. However, complement originating from the peripheral circulation can enter the brain either through a compromised blood-brain barrier or via the naturally highly permeable meningeal barrier, potentially influencing CNS function (125). A key limitation is that our mechanistic interpretation cannot be confirmed because we did not include direct CNS measures. Specifically, we did not assess neuroimaging correlates of brain structure/inflammation or measure complement components in cerebrospinal fluid (CSF). Therefore, links between peripheral complement levels and central immune activation, immune-mediated neural injury, or excessive synaptic pruning remain inferential and should be tested in future multimodal studies.

Fifth, medication and lifestyle effects were not assessed systematically, and the study was not powered to evaluate these influences in detail; therefore, residual confounding cannot be excluded. Nevertheless, exploratory analyses did not indicate statistically significant effects of age, sex, smoking status, BMI, or antipsychotic exposure (treatment status and CPZ equivalents) on baseline C3/C4 levels, and C3/C4 concentrations did not differ significantly between patients treated vs not treated at admission, with small effect sizes.

Finally, childhood trauma was assessed using self-report questionnaires, which may be subject to recall bias. In addition, we did not assess complement genetic variation (e.g., C4A/C4B copy number), which may influence peripheral complement levels and clinical associations.

Despite these limitations, the exploratory nature of this study provides novel insights into the potential role of complement-related immune processes in schizophrenia and highlights the need for larger, controlled longitudinal studies to confirm and extend these findings.

## 7 Conclusions

Overall, this study identified several nominal, exploratory within-cohort associations between baseline serum complement markers (C3/C4) and clinical features in schizophrenia; however, none of the associations survived BH-FDR correction for multiple comparisons (all q>0.05). In this cohort of inpatients undergoing psychotic decompensation, higher baseline serum C3 levels were nominally associated with a longer duration of untreated psychosis and a longer length of hospitalization, and were nominally related to greater symptom severity at admission. Baseline C4 levels were nominally associated with general psychopathology at admission and were nominally related to higher symptom severity across multiple PANSS dimensions at the 12-week follow-up (Positive, Negative, General, and Total PANSS scores). Taken together, these nominal findings highlight potential relationships between interindividual differences in complement markers at admission and variability in symptom burden at admission and follow-up, while C3 additionally showed nominal links with illness-course indicators in this cohort.

Furthermore, to the best of our knowledge, this is the first study to report nominal associations between greater exposure to childhood trauma and higher baseline peripheral levels of C3 and C4 in patients with schizophrenia. In addition, baseline complement levels were nominally related to anxiety severity both at admission and after 12 weeks of inpatient treatment. These observations are consistent with the hypothesis that complement system activation may be linked to early-life trauma and stress-related vulnerability in schizophrenia, potentially reflecting bidirectional processes in which higher complement levels relate to neuroinflammation and synaptic dysregulation relevant to anxiety symptoms, while trauma history and anxiety-related stress may further amplify immune activation. These exploratory results motivate further research on the role of complement-related immune dysregulation in anxiety and symptom burden in schizophrenia. They also highlight the need to examine how childhood trauma and anxiety-related stress may relate to inflammatory activity over time.

This study also identified a nominal association between complement markers and cognitive functioning in schizophrenia, with lower baseline C3 levels being nominally associated with better cognitive performance at hospital admission. Overall, baseline C3 may be explored as a peripheral correlate of cognitive performance in schizophrenia, although confirmation in larger longitudinal studies with repeated complement measurements and more detailed neuropsychological testing is warranted. In line with prior evidence (e.g., Yu et al., 2023) (58), the present results add to emerging data linking peripheral complement components to cognitive phenotypes in schizophrenia. Given the potential adverse effects of complement dysregulation on CNS function, complement-related pathways may warrant further investigation as candidate targets for interventions addressing cognitive deficits; however, mechanistic studies and adequately powered clinical trials will be required to establish causality and therapeutic relevance.

Taken together, these nominal, exploratory findings highlight complement system involvement across symptom severity, illness-course indicators, anxiety, and cognitive function in schizophrenia, while underscoring the need for replication. Future studies should test whether these associations are reproducible after multiplicity correction, and whether complement-related pathways prospectively track longitudinal outcomes and clinically meaningful subgroups in larger, controlled designs.

## 8 Future directions

Further research should integrate peripheral complement markers (C3/C4) with neuroimaging by combining structural magnetic resonance imaging (MRI) (e.g., cortical thickness, hippocampal volume) and magnetic resonance spectroscopy (MRS) to test whether complement alterations relate to central neuroinflammation and synaptic loss in schizophrenia.

Longitudinal designs are needed to track peripheral complement markers (C3/C4) from the clinical high-risk (CHR) phase through first-episode psychosis and into remission to establish the temporal dynamics of complement alterations across illness stages.

Intervention studies, including clinical trials, should evaluate whether pharmacological modulation of the complement system—using complement inhibitors such as C1 esterase inhibitors or C5a receptor antagonists—can reduce symptom severity and improve cognitive outcomes in schizophrenia.

In parallel, a broader neuroinflammatory profiling approach should assess complement markers alongside additional immune measures, including cytokines, complement activation fragments (C3a, C4a), soluble terminal complement complex (sTCC), and C-reactive protein, to identify immune subtypes and develop informative multimarker biomarker combinations.

Finally, mechanistic MRS analyses should test whether peripheral complement markers (C3/C4) are associated with key metabolites, including N-acetylaspartate (NAA) as an index of neuronal integrity, myo-inositol as a marker of glial activation, and glutamate measures reflecting glutamatergic homeostasis.

## Data Availability

The datasets presented in this article are not readily available because they contain sensitive clinical data from human participants and cannot be made publicly available due to ethical and privacy restrictions. Requests to access the datasets should be directed to the corresponding author.
