# Supplementary material for: Peripheral complement C3 and C4 are associated with clinical features of schizophrenia
Source: Front Psychiatry. 2026 Mar 30;17:1767438. doi: 10.3389/fpsyt.2026.1767438 (PMC13071058; doi:10.3389/fpsyt.2026.1767438)
Supplement: Supplementary file 1 [file Table1.docx]

**Supplementary Table S1.** **Spearman correlations between C3 concentration and analysed parameters**

| Features | Size | r_s_ | p-value  uncorrected | q-value (BH-FDR adjusted) |
| --- | --- | --- | --- | --- |
| Age [years] | 39 | -0.22 | 0.186 | 0.330 |
| Age of first episode of psychosis [years] | 39 | -0.15 | 0.350 | 0.491 |
| BMI - T1 [kg/m^2^] | 39 | -0.12 | 0.464 | 0.603 |
| BMI - T2 [kg/m^2^] | 39 | -0.08 | 0.646 | 0.744 |
| BMI ΔT (T2 - T1) [kg/m^2^] | 39 | 0.14 | 0.403 | 0.583 |
| Duration of untreated psychosis [days] | 39 | 0.41 | 0.010 | 0.156 |
| Length of hospitalization [days] | 39 | 0.35 | 0.028 | 0.163 |
| Number of psychosis episodes | 39 | -0.17 | 0.297 | 0.482 |
| Duration of illness [days] | 39 | -0.02 | 0.909 | 0.909 |
| PANSS-P_1 | 39 | 0.33 | 0.043 | 0.163 |
| PANSS-N_1 | 39 | 0.16 | 0.329 | 0.501 |
| PANSS-G_1 | 39 | 0.33 | 0.040 | 0.163 |
| PANSS-Total_1 | 39 | 0.23 | 0.166 | 0.300 |
| PANSS-P_2 | 39 | 0.13 | 0.436 | 0.603 |
| PANSS-N_2 | 39 | -0.02 | 0.892 | 0.909 |
| PANSS-G_2 | 39 | 0.05 | 0.790 | 0.861 |
| PANSS-Total_2 | 39 | 0.23 | 0.166 | 0.300 |
| PANSS-P ΔT (T2 - T1) | 39 | -0.32 | 0.054 | 0.176 |
| PANSS-N ΔT (T2 - T1) | 39 | 0.04 | 0.817 | 0.861 |
| PANSS-G ΔT (T2 - T1) | 39 | -0.26 | 0.117 | 0.268 |
| PANSS-Total ΔT (T2 - T1) | 39 | -0.25 | 0.136 | 0.294 |
| MoCA-1 | 39 | -0.34 | 0.034 | 0.163 |
| MoCA-2 | 39 | -0.29 | 0.079 | 0.200 |
| MoCA ΔT (T2 - T1) | 39 | -0.10 | 0.567 | 0.691 |
| STAI-T_1 | 39 | 0.38 | 0.018 | 0.152 |
| STAI-S_1 | 39 | 0.37 | 0.020 | 0.156 |
| STAI-T_2 | 39 | 0.38 | 0.018 | 0.152 |
| STAI-S_2 | 39 | 0.42 | 0.009 | 0.156 |
| STAI-T ΔT (T2 - T1) | 39 | -0.23 | 0.159 | 0.308 |
| STAI-S ΔT (T2 - T1) | 39 | 0.10 | 0.543 | 0.683 |
| CTQ_EN | 39 | 0.18 | 0.266 | 0.444 |
| CTQ_EA | 39 | 0.32 | 0.046 | 0.163 |
| CTQ_PN | 39 | 0.17 | 0.315 | 0.491 |
| CTQ_PA | 39 | 0.29 | 0.074 | 0.205 |
| CTQ_SA | 39 | 0.30 | 0.068 | 0.204 |
| CTQ_Total | 39 | 0.34 | 0.034 | 0.163 |
| Chlorpromazine-equivalent dose - baseline | 39 | 0.04 | 0.797 | 0.861 |
| Chlorpromazine-equivalent dose - week 12 | 39 | 0.27 | 0.100 | 0.244 |

*Features - analysed variables; rs - Spearman correlation coefficient; p - uncorrected p-value; q-values - p-values adjusted for multiple testing using the Benjamini-Hochberg false discovery rate (BH-FDR) procedure.*
