# Supplementary material for: Peripheral complement C3 and C4 are associated with clinical features of schizophrenia
Source: Front Psychiatry. 2026 Mar 30;17:1767438. doi: 10.3389/fpsyt.2026.1767438 (PMC13071058; doi:10.3389/fpsyt.2026.1767438)
Supplement: Supplementary file 2 [file Table2.docx]

**Supplementary Table S2. Spearman correlations between C4 concentration and analysed parameters**

| Features | Size | r_s_ | p-value  uncorrected | q-value (BH-FDR adjusted) |
| --- | --- | --- | --- | --- |
| Age [years] | 39 | 0.11 | 0.522 | 0.764 |
| BMI - T1 [kg/m^2^] | 39 | -0.17 | 0.286 | 0.567 |
| BMI - T2 [kg/m^2^] | 39 | -0.08 | 0.637 | 0.756 |
| BMI ΔT (T2 - T1) [kg/m^2^] | 39 | 0.20 | 0.230 | 0.498 |
| Age of first episode of psychosis [years] | 39 | 0.15 | 0.358 | 0.582 |
| Duration of untreated psychosis [days] | 39 | 0.32 | 0.051 | 0.165 |
| Length of hospitalization [days] | 39 | 0.13 | 0.419 | 0.653 |
| Number of psychosis episodes | 39 | 0.00 | 0.993 | 0.993 |
| Duration of illness [days] | 39 | 0.16 | 0.341 | 0.582 |
| PANSS-P_1 | 39 | 0.28 | 0.085 | 0.215 |
| PANSS-N_1 | 39 | 0.17 | 0.313 | 0.582 |
| PANSS-G_1 | 39 | 0.32 | 0.045 | 0.159 |
| PANSS-Total_1 | 39 | 0.28 | 0.088 | 0.215 |
| PANSS-P_2 | 39 | 0.38 | 0.018 | 0.140 |
| PANSS-N_2 | 39 | 0.38 | 0.018 | 0.140 |
| PANSS-G_2 | 39 | 0.40 | 0.014 | 0.140 |
| PANSS-Total_2 | 39 | 0.37 | 0.018 | 0.140 |
| PANSS-P ΔT (T2 - T1) | 39 | -0.09 | 0.610 | 0.756 |
| PANSS-N ΔT (T2 - T1) | 39 | 0.11 | 0.503 | 0.752 |
| PANSS-G ΔT (T2 - T1) | 39 | -0.04 | 0.805 | 0.872 |
| PANSS-Total ΔT (T2 - T1) | 39 | -0.01 | 0.965 | 0.993 |
| MoCA-1 | 39 | -0.07 | 0.659 | 0.760 |
| MoCA-2 | 39 | -0.11 | 0.509 | 0.752 |
| MoCA ΔT (T2 - T1) | 39 | -0.05 | 0.768 | 0.856 |
| STAI-T_1 | 39 | 0.36 | 0.024 | 0.156 |
| STAI-S_1 | 39 | 0.33 | 0.033 | 0.159 |
| STAI-T_2 | 39 | 0.39 | 0.055 | 0.165 |
| STAI-S_2 | 39 | 0.34 | 0.038 | 0.159 |
| STAI-T ΔT (T2 - T1) | 39 | -0.08 | 0.651 | 0.756 |
| STAI-S ΔT (T2 - T1) | 39 | -0.08 | 0.621 | 0.756 |
| CTQ_EN | 39 | 0.28 | 0.088 | 0.215 |
| CTQ_EA | 39 | 0.32 | 0.043 | 0.159 |
| CTQ_PN | 39 | 0.12 | 0.478 | 0.719 |
| CTQ_PA | 39 | 0.24 | 0.144 | 0.330 |
| CTQ_SA | 39 | 0.40 | 0.011 | 0.140 |
| CTQ_Total | 39 | 0.32 | 0.044 | 0.159 |
| Chlorpromazine-equivalent dose - baseline | 39 | 0.01 | 0.972 | 0.993 |
| Chlorpromazine-equivalent dose - week 12 | 39 | 0.17 | 0.291 | 0.567 |

*Features - analysed variables; rs - Spearman correlation coefficient; p - uncorrected p-value; q-values - p-values adjusted for multiple testing using the Benjamini-Hochberg false discovery rate (BH-FDR) procedure.*
