# Supplementary material for: Peripheral complement C3 and C4 are associated with clinical features of schizophrenia
Source: Front Psychiatry. 2026 Mar 30;17:1767438. doi: 10.3389/fpsyt.2026.1767438 (PMC13071058; doi:10.3389/fpsyt.2026.1767438)
Supplement: Supplementary file 3 [file Table3.docx]

**Supplementary Table S3. Comparison of groups with low and high C3 levels (according to median value) in terms of the analysed parameters using Mann-Whitney test**

| Features* | C3 ≤ median value 1.4 g/L (n=22) | C3 > median value 1.4 g/L (n=17) | *P value* | Effect size (Cliff’s δ) |
| --- | --- | --- | --- | --- |
|  | Median value, Q1, Q3 | Median value, Q1, Q3 |  |  |
| Age [years] | 22.0, 17.0, 32.0 | 17.0, 17.0, 23.0 | 0.257 | 0.217 |
| BMI - T1 [kg/m^2^] | 21.2, 19.9, 24.2 | 20.8, 19.5, 22.1 | 0.768 | 0.059 |
| BMI - T2 [kg/m^2^] | 22.1, 19.9, 24.4 | 22.3, 21.4, 24.0 | 0.643 | -0.088 |
| BMI ΔT (T2 - T1) [kg/m^2^] | 1.0, 0.0, 1.4 | 1.3, 0.5, 1.8 | 0.408 | -0.160 |
| Age of first episode of psychosis [years] | 17.5, 16.0, 21.0 | 17.0, 16.0, 20.0 | 0.967 | 0.008 |
| Duration of untreated psychosis [days] | 12.0, 7.0, 21.0 | 21.0, 7.0, 56.0 | 0.055 | -0.361 |
| Length of hospitalization [days] | 53.5, 34.0, 60.0 | 68.0, 50.0, 97.0 | 0.104 | -0.307 |
| Number of psychosis episodes | 1.5, 1.0, 7.0 | 1.0, 1.0, 2.0 | 0.119 | 0.262 |
| Duration of illness [days] | 0.6, 0.2, 12.0 | 1.6, 0.2, 1.0 | 0.172 | -0.094 |
| PANSS-P_1 | 25.5, 21.0, 33.0 | 31.0, 26.0, 34.0 | 0.092 | -0.318 |
| PANSS-N_1 | 26.0, 23.0, 30.0 | 26.0, 24.0, 28.0 | 0.994 | 0.013 |
| PANSS-G_1 | 51.0, 46.0, 61.0 | 61.0, 53.0, 65.0 | 0.092 | -0.318 |
| PANSS-Total_1 | 97.0, 88.0, 123.0 | 117.0, 106.0, 127.0 | 0.292 | -0.203 |
| PANSS-P_2 | 12.0, 9.0, 13.0 | 11.0, 10.0, 16.0 | 0.728 | 0.112 |
| PANSS-N_2 | 15.0, 13.0, 19.0 | 14.0, 11.0, 21.0 | 0.712 | 0.112 |
| PANSS-G_2 | 28.0, 27.0, 33.0 | 30.0, 28.0, 35.0 | 0.718 | -0.071 |
| PANSS-Total_2 | 53.5, 48.0, 63.0 | 62.0, 51.0, 70.0 | 0.319 | -0.193 |
| PANSS-P ΔT (T2 - T1) | -13.0, -16.0, -10.0 | -16.0, -22.0, -13.0 | 0.056 | 0.393 |
| PANSS-N ΔT (T2 - T1) | -11.0, -13.0, -7.0 | -10.0, -13.0, -4.0 | 0.816 | -0.088 |
| PANSS-G ΔT (T2 - T1) | -22.0, -28.0, -14.0 | -28.0, -32.0, -18.0 | 0.121 | 0.332 |
| PANSS-Total ΔT (T2 - T1) | -41.0, -58.0, -36.0 | -49.0, -68.0, -39.0 | 0.136 | 0.318 |
| MoCA-1 | 16.5, 14.0, 19.0 | 13.0, 12.0, 17.0 | 0.039 | 0.390 |
| MoCA-2 | 23.0, 19.0, 25.0 | 19.0, 15.0, 22.0 | 0.048 | 0.406 |
| MoCA ΔT (T2 - T1) | 5.0, 3.0, 6.0 | 4.0, 3.0, 6.0 | 0.581 | 0.150 |
| STAI-T_1 | 57.0, 51.0, 60.0 | 64.0, 58.0, 68.0 | 0.036 | -0.396 |
| STAI-S_1 | 55.0, 49.0, 59.0 | 59.0, 55.0, 65.0 | 0.077 | -0.337 |
| STAI-T_2 | 49.0, 46.0, 52.0 | 52.0, 50.0, 55.0 | 0.045 | -0.412 |
| STAI-S_2 | 47.0, 44.0, 50.0 | 50.0, 48.0, 53.0 | 0.036 | -0.425 |
| STAI-T ΔT (T2 - T1) | -7.0, -8.0, -5.0 | -9.0, -10.0, -7.0 | 0.161 | 0.302 |
| STAI-S ΔT (T2 - T1) | -7.0, -10.0, -5.0 | -7.0, -10.0, -4.0 | 0.542 | -0.158 |
| CTQ_EN | 15.0, 14.0, 19.0 | 19.0, 15.0, 21.0 | 0.138 | -0.281 |
| CTQ-EA | 12.0, 10.0, 14.0 | 16.0, 13.0, 19.0 | 0.023 | -0.425 |
| CTQ_PN | 10.0, 8.0, 13.0 | 12.0, 9.0, 16.0 | 0.243 | -0.222 |
| CTQ_PA | 7.0, 6.0, 9.0 | 10.0, 8.0, 14.0 | 0.017 | -0.449 |
| CTQ_SA | 5.0, 5.0, 5.0 | 5.0, 5.0, 6.0 | 0.474 | -0.139 |
| CTQ-Total | 63.0, 53.0, 69.0 | 86.0, 60.0, 89.0 | 0.027 | -0.414 |
| Chlorpromazine Equivalent Dose - Baseline | 100.0, 0.0, 150.0 | 100.0, 0.0, 100.0 | 0.705 | 0.075 |
| Chlorpromazine Equivalent Dose - Week 12 | 400.0, 300.0, 600.0 | 533.3, 400.0, 600.0 | 0.098 | -0.316 |

- *For all variables presented in this table, data are available from 39 patients*
