# Supplementary material for: Peripheral complement C3 and C4 are associated with clinical features of schizophrenia
Source: Front Psychiatry. 2026 Mar 30;17:1767438. doi: 10.3389/fpsyt.2026.1767438 (PMC13071058; doi:10.3389/fpsyt.2026.1767438)
Supplement: Supplementary file 4 [file Table4.docx]

**Supplementary Table S4. Comparison of groups with low and high C4 levels (according to median value) in terms of the analysed parameters using Mann-Whitney test**

| Features* | C4 ≤ median value  0.2 g/L (n=22) | C4 > median value  0.2 g/L (n=17) | *P value* | Effect size (Cliff’s δ) |
| --- | --- | --- | --- | --- |
|  | Median value, Q1, Q3 | Median value, Q1, Q3 |  |  |
| Age [years] | 18.0, 16.0, 27.0 | 21.0, 10.0, 29.0 | 0.392 | -0.166 |
| BMI - T1 [kg/m^2^] | 21.2, 19.5, 26.5 | 20.5, 19.9, 22.1 | 0.333 | 0.187 |
| BMI - T2 [kg/m^2^] | 22.1, 20.1, 25.6 | 22.3, 21.0, 23.9 | 0.878 | -0.032 |
| BMI ΔT (T2 - T1) [kg/m^2^] | 0.8, 0.0, 1.3 | 1.4, 0.8, 1.6 | 0.081 | -0.332 |
| Age of first episode of psychosis [years] | 17.0, 16.0, 19.0 | 19.0, 17.0, 23.0 | 0.106 | -0.305 |
| Duration of untreated psychosis [days] | 14.0, 7.0, 21.0 | 14.0, 7.0, 56.0 | 0.492 | -0.131 |
| Length of hospitalization [days] | 55.5, 34.0, 68.0 | 62.0, 49.0, 83.0 | 0.362 | -0.174 |
| Number of psychosis episodes | 1.0, 1.0, 5.0 | 1.0, 1.0, 5.0 | 0.110 | 0.067 |
| Duration of illness [days] | 0.3, 0.1, 6.0 | 0.4, 0.2, 5.0 | 0.702 | -0.075 |
| PANSS-P_1 | 27.0, 21.0, 32.0 | 33.0, 24.0, 35.0 | 0.321 | -0.289 |
| PANSS-N_1 | 24.5, 20.0, 29.0 | 27.0, 25.0, 29.0 | 0.120 | -0.243 |
| PANSS-G_1 | 53.0, 43.0, 61.0 | 61.0, 47.0, 65.0 | 0.110 | -0.302 |
| PANSS-Total_1 | 99.0, 86.0, 123.0 | 118.0, 96.0, 130.0 | 0.124 | -0.294 |
| PANSS-P_2 | 10.5, 9.0, 13.0 | 13.0, 10.0, 16.0 | 0.084 | -0.374 |
| PANSS-N_2 | 13.0, 11.0, 16.0 | 18.5, 13.5, 21.0 | 0.036 | -0.436 |
| PANSS-G_2 | 28.0, 25.0, 32.0 | 33.5, 28.5, 37.0 | 0.017 | -0.487 |
| PANSS-Total_2 | 51.5, 48.0, 62.0 | 64.0, 52.0, 70.0 | 0.029 | -0.409 |
| PANSS-P ΔT (T2 - T1) | -13.5, -20.0, -10.0 | -16.0, -19.0, -12.0 | 0.609 | 0.152 |
| PANSS-N ΔT (T2 - T1) | -10.0, -14.0, -7.0 | -11.0, -13.0, -4.0 | 0.510 | 0.182 |
| PANSS-G ΔT (T2 - T1) | -24.5, -28.0, -16.0 | -24.5, -30.5, -16.0 | 0.804 | 0.104 |
| PANSS-Total ΔT (T2 - T1) | -42.0, -61.0, -37.0 | -48.0, -64.0, -37.0 | 0.965 | 0.067 |
| MoCA-1 | 15.0, 12.0, 18.0 | 15.0, 13.0, 19.0 | 0.685 | -0.078 |
| MoCA-2 | 20.5, 16.0, 24.0 | 21.5, 16.5, 23.5 | 0.941 | -0.072 |
| MoCA ΔT (T2 - T1) | 4.5, 3.0, 6.0 | 4.0, 3.5, 5.5 | 0.781 | 0.112 |
| STAI-T_1 | 59.5, 50.0, 63.0 | 60.0, 55.0, 68.0 | 0.267 | -0.211 |
| STAI-S_1 | 55.0, 49.0, 61.0 | 59.0, 53.0, 65.0 | 0.221 | -0.233 |
| STAI-T_2 | 50.0, 46.0, 52.0 | 50.0, 48.5, 55.0 | 0.298 | -0.251 |
| STAI-S_2 | 47.0, 44.0, 51.0 | 49.0, 47.5, 53.0 | 0.201 | -0.291 |
| STAI-T ΔT (T2 - T1) | -8.0, -10.0, -5.0 | -8.5, -10.0, -5.0 | 0.651 | 0.141 |
| STAI-S ΔT (T2 - T1) | -7.0, -10.0, -4.0 | -7.0, -12.0, -4.5 | 0.630 | 0.150 |
| CTQ_EN | 14.5, 14.0, 19.0 | 19.0, 16.0, 21.0 | 0.292 | -0.201 |
| CTQ-EA | 13.0, 11.0, 15.0 | 15.0, 10.0, 18.0 | 0.457 | -0.144 |
| CTQ_PN | 11.0, 8.0, 15.0 | 10.0, 8.0, 16.0 | 0.997 | 0.008 |
| CTQ_PA | 8.0, 6.0, 10.0 | 8.0, 6.0, 12.0 | 0.547 | -0.115 |
| CTQ_SA | 5.0, 5.0, 5.0 | 5.0, 5.0, 6.0 | 0.255 | -0.217 |
| CTQ-Total | 63.5, 55.0, 85.0 | 66.0, 63.0, 90.0 | 0.362 | -0.176 |
| Chlorpromazine Equivalent Dose - Baseline | 100.0, 0.0, 150.0 | 100.0, 100.0, 100.0 | 0.474 | -0.136 |
| Chlorpromazine Equivalent Dose - Week 12 | 400.0, 300.0, 600.0 | 400.0, 400.0, 800.0 | 0.292 | -0.203 |

*For all variables presented in this table, data are available from 39 patients
