# Supplementary material for: Peripheral complement C3 and C4 are associated with clinical features of schizophrenia
Source: Front Psychiatry. 2026 Mar 30;17:1767438. doi: 10.3389/fpsyt.2026.1767438 (PMC13071058; doi:10.3389/fpsyt.2026.1767438)
Supplement: Supplementary file 5 [file Table5.docx]

**Supplementary Table S5. Complement C3 and C4 concentrations by treatment status (Mann-Whitney U test)**

| Variable (g/L) | Treatment | | p-value | Effect size (Cliff’s δ) |
| --- | --- | --- | --- | --- |
|  | No | Yes |  |  |
| C3, median (Q1–Q3) | 1.22, 1.11, 1.53 | 1.39, 1.11, 1.79 | 0.590 | 0.117 |
| C4, median (Q1–Q3) | 0.22, 0.16, 0.22 | 0.24, 0.18, 0.28 | 0.331 | 0.208 |

Treatment status refers to antipsychotic treatment at admission (yes/no) (N_no=11, N_yes=28).
