# Supplementary material for: Peripheral complement C3 and C4 are associated with clinical features of schizophrenia
Source: Front Psychiatry. 2026 Mar 30;17:1767438. doi: 10.3389/fpsyt.2026.1767438 (PMC13071058; doi:10.3389/fpsyt.2026.1767438)
Supplement: Supplementary file 6 [file Table6.docx]

**Supplementary Table S6. Comparison of the current study with recent key studies assessing peripheral complement markers in schizophrenia**

| **STUDY** | **SAMPLE / STAGE** | **AGE** | **DESIGN / TIMEPOINTS** | **COMPLEMENT MEASURE (MATRIX; ASSAY)** | **OUTCOMES ASSESSED** | **MAIN FINDINGS** |
| --- | --- | --- | --- | --- | --- | --- |
| **Current study (Szwajca et al.)** | n=39; inpatient Sz in psychotic decompensation; 28% unmedicated at admission; no controls | mean age of 22.4 ± 1.2 years (SE). | Prospective within-cohort study; baseline serum C3/C4 measured at admission (T1) with clinical follow-up after 12 weeks of inpatient treatment (T2). | C3 (C3c) & C4 (C4c) (serum; immunonephelometry; morning fasting samples at admission) | **PANSS** (Positive and Negative Syndrome Scale), **STAI (State–Trait Anxiety** Inventory), **MoCA** (Montreal Cognitive Assessment; baseline and 12-week follow-up), **CTQ** (Childhood Trauma Questionnaire), DUP (duration of untreated psychosis), and **hospitalization length** (length of hospital stay). | Following Benjamini–Hochberg FDR correction, none of the C3/C4 correlations with clinical variables were statistically significant (all q>0.05). In exploratory analyses based on nominal (uncorrected) p-values, C3 nominally correlated with DUP and hospitalization length, and with higher PANSS (baseline), higher STAI (baseline & 12w), higher CTQ, and lower MoCA (baseline). C4 nominally correlated with PANSS (baseline and/or 12w) and STAI (baseline & 12w) and CTQ. |
| **Göker et al., 2023 (56)** | Schizophrenia in remission (n=53), treatment-resistant schizophrenia (n=53), healthy controls (n=53) | 40.11 ± 10.40 years (no significant age difference between the three groups; p = 0.933) | Cross-sectional, single assessment (no follow-up timepoints reported) | C3 & C4 (serum; nephelometry) — after 12-h overnight fasting, serum separator tubes; measured on Beckman Array Protein System | Symptom severity & clinical status: **BPRS** (Brief Psychiatric Rating Scale), **PANSS** (Positive and Negative Syndrome Scale), **CGI** (Clinical Global Impression); functioning: **GAF** (Global Assessment of Functioning); cognitive insight: **BCIS** (Beck Cognitive Insight Scale) | C3 and C4 were higher in treatment-resistant schizophrenia vs remission and controls. Both complements showed positive correlations with symptom severity (PANSS/CGI-S; C4 also with BPRS) and negative correlations with functioning (GAF). In multivariable analysis, higher C4 increased odds of treatment resistance (OR≈1.13). |
| **Cao et al., 2023 (57)** | 89 first-episode, drug-naïve schizophrenia patients + 89 age/sex/ethnicity-matched healthy controls | 35.96 ± 12.65 years in the FES (SCZ) group and 35.78 ± 11.05 years in healthy controls. | Cross-sectional case–control; single assessment (one timepoint) | C1, C2, C3, C4 and CH50 (serum; ELISA; blood drawn 7:00–8:00 a.m.). | Symptom severity:  **BPRS-18** (Brief Psychiatric Rating Scale, 18-item version), **SANS** (Scale for the Assessment of Negative Symptoms), **SAPS** (Scale for the Assessment of Positive Symptoms);  diagnostic performance: **ROC/AUC (**Receiver Operating Characteristic / Area Under the Curve) | Serum C3 and C4 levels were increased in first-episode, drug-naïve schizophrenia vs healthy controls. C3 showed a positive correlation with SAPS (symptom severity), whereas C4 showed no key symptom correlation. In ROC analyses, C3 (AUC≈0.668) and C4 (AUC≈0.656) each had limited standalone discriminative value (AUC < 0.7) |
| **Yu et al., 2023 (58)** | Drug-naïve first-episode schizophrenia (n=52) vs healthy controls (n=52) | FES: 21.71 ± 7.16; HC: 27.65 ± 8.97 years | Cross-sectional, single timepoint | Complement panel (C1q, C3, C3b/iC3b, C4, factor B, factor H and properdin) (EDTA plasma; multiplex immunoassay; blood drawn 4:00–4:30 p.m.). | Cortical thickness (MRI), logical memory (immediate & delayed recall), symptom severity (PANSS) | In FES, higher C3 was associated with worse delayed logical memory, and higher C4 with thinner left primary sensory cortex; no C3/C4–PANSS associations. Across all participants (FES+HC), higher C4 was associated with worse immediate logical memory. |
| **Savukoski et al., 2024 (59)** | Acutely ill, unmedicated schizophrenia patients (n = 96), including first-episode schizophrenia (FESz) patients (n = 61) who were antipsychotic-naïve, and relapsed schizophrenia (RSz) patients (n = 35) who had been unmedicated for ≥ 6 weeks, plus matched healthy controls (n = 96); follow-up data were available for n = 57 patients. | Sz 33.0 [26.3–44.5] vs controls 34.5 [27.0–45.8] years. | Longitudinal case–control: baseline (T0) + 6-week follow-up (T6) after antipsychotic treatment. | Fasting 08:00 a.m.; C4 (serum cleavage marker; ELISA) plus terminal activation marker sTCC (plasma; ELISA); C3 not assessed. | PANSS (baseline & 6w); inflammatory/metabolic covariates incl. CRP, BMI, smoking (as confounders/covariates). | sTCC was elevated in schizophrenia vs controls at baseline and did not change after 6 weeks of antipsychotic treatment; no association with symptom severity (PANSS) was found. C4 showed no case–control difference, no change over 6 weeks, and no association with symptom severity; sTCC associated with CRP. |
