# Supplementary figures and images for: Peripheral complement C3 and C4 are associated with clinical features of schizophrenia
Source: Front Psychiatry. 2026 Mar 30;17:1767438. doi: 10.3389/fpsyt.2026.1767438 (PMC13071058; doi:10.3389/fpsyt.2026.1767438)

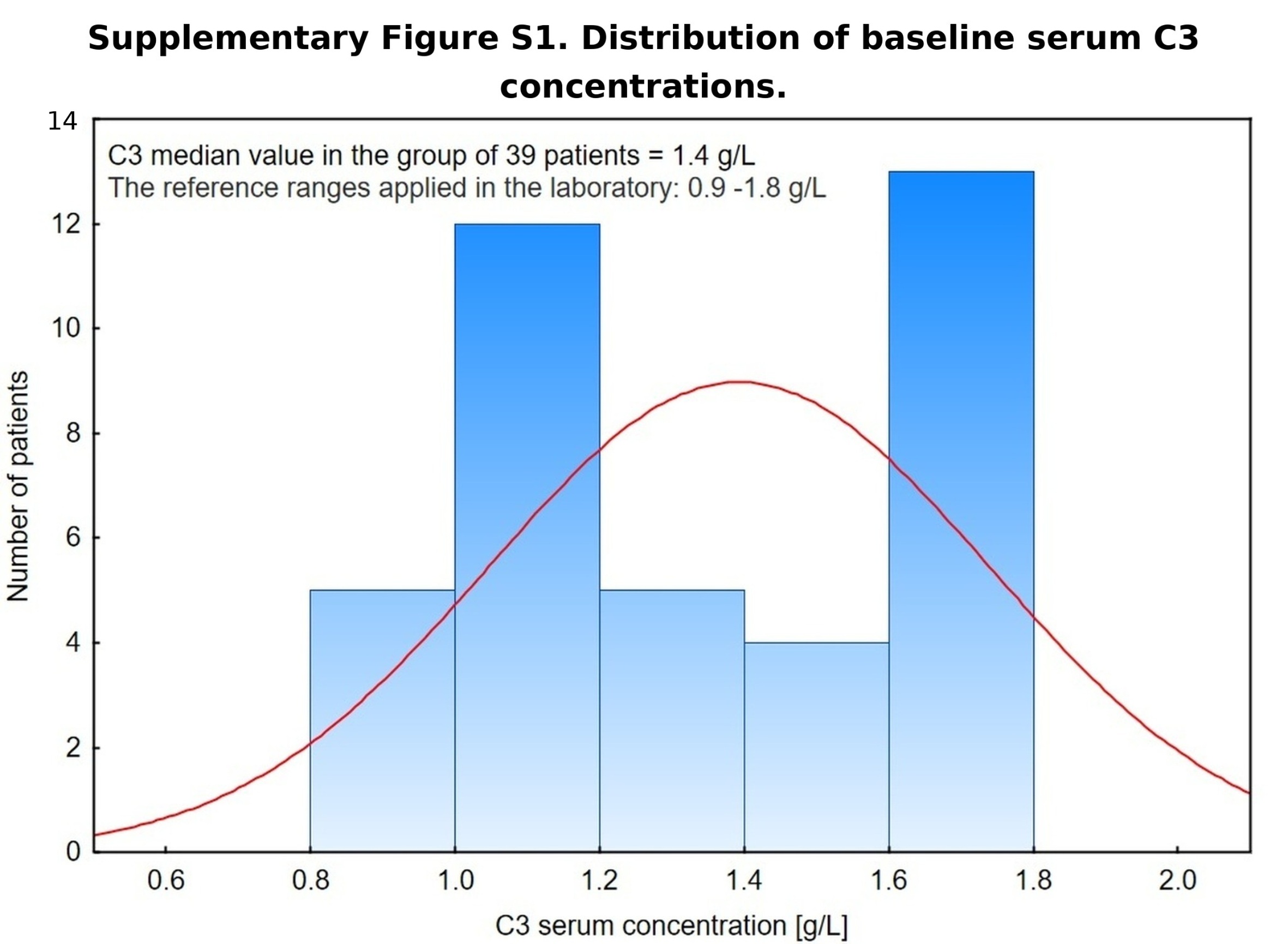

Supplement: Supplementary file 7 [file Image1.jpeg]

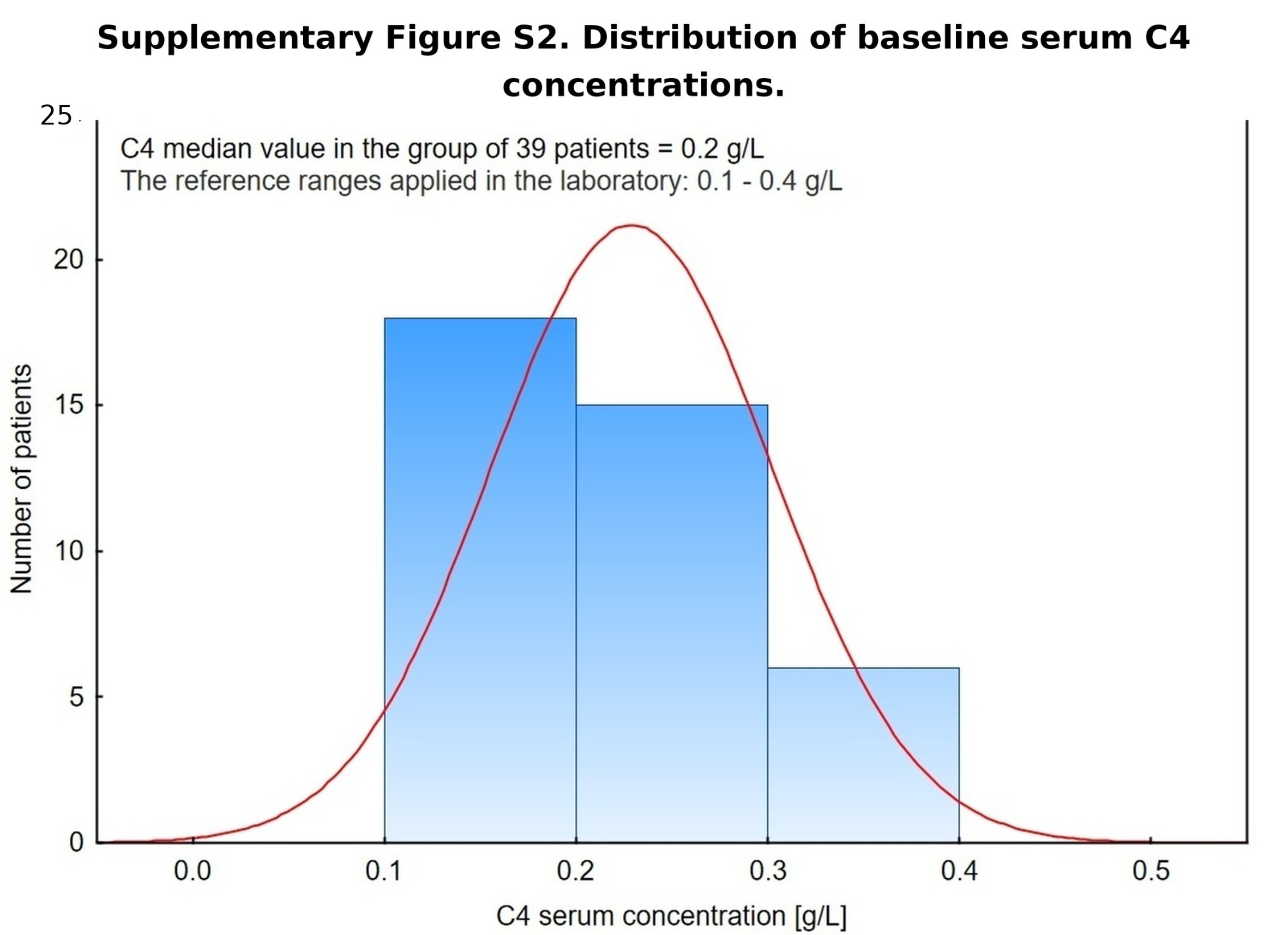

Supplement: Supplementary file 8 [file Image2.jpeg]
